# Incorporation of the histone variant H2A.Z counteracts gene silencing mediated by H3K27 trimethylation in *Fusarium fujikuroi*

**DOI:** 10.1186/s13072-024-00532-y

**Published:** 2024-03-20

**Authors:** Anna K. Atanasoff-Kardjalieff, Harald Berger, Katharina Steinert, Slavica Janevska, Nadia Ponts, Hans-Ulrich Humpf, Svetlana Kalinina, Lena Studt-Reinhold

**Affiliations:** 1 Department of Applied Genetics and Cell Biology, Institute of Microbial Genetics, University of Natural Resources and Life Sciences, Vienna, Konrad-Lorenz Strasse 24, Tulln an der Donau, 3430 Austria; 2 Institute of Food Chemistry, University of Münster, Corrensstraße 45, 48149 Münster, Germany; 3 (Epi-)Genetic Regulation of Fungal Virulence, Leibniz Institute for Natural Product Research and Infection Biology–Hans Knöll Institute, 07745 Jena, Germany; 4 INRAE, UR1264 Mycology and Food Safety (MycSA), Villenave d’Ornon, 33882 France

**Keywords:** Histone variant H2A.Z, Chromatin, *Fusarium*, Gene regulation, Transcription

## Abstract

**Background:**

*Fusarium fujikuroi* is a pathogen of rice causing diverse disease symptoms such as ‘*bakanae*’ or stunting, most likely due to the production of various natural products (NPs) during infection. Fusaria have the genetic potential to synthesize a plethora of these compounds with often diverse bioactivity. The capability to synthesize NPs exceeds the number of those being produced by far, implying a gene regulatory network decisive to induce production. One such regulatory layer is the chromatin structure and chromatin-based modifications associated with it. One prominent example is the exchange of histones against histone variants such as the H2A variant H2A.Z. Though H2A.Z already is well studied in several model organisms, its regulatory functions are not well understood. Here, we used *F. fujikuroi* as a model to explore the role of the prominent histone variant FfH2A.Z in gene expression within euchromatin and facultative heterochromatin.

**Results:**

Through the combination of diverse ‘-omics‘ methods, we show the global distribution of FfH2A.Z and analyze putative crosstalks between the histone variant and two prominent histone marks, i.e., H3K4me3 and H3K27me3, important for active gene transcription and silencing, respectively. We demonstrate that, if FfH2A.Z is positioned at the + 1-nucleosome, it poises chromatin for gene transcription, also within facultative heterochromatin. Lastly, functional characterization of *FfH2A.Z* overexpression and depletion mutants revealed that FfH2A.Z is important for wild type-like fungal development and secondary metabolism.

**Conclusion:**

In this study, we show that the histone variant FfH2A.Z is a mark of positive gene transcription and acts independently of the chromatin state most likely through the stabilization of the + 1-nucleosome. Furthermore, we demonstrate that FfH2A.Z depletion does not influence the establishment of both H3K27me3 and H3K4me3, thus indicating no crosstalk between FfH2A.Z and both histone marks. These results highlight the manifold functions of the histone variant FfH2A.Z in the phytopathogen *F. fujikuroi*, which are distinct regarding gene transcription and crosstalk with the two prominent histone marks H3K27me3 and H3K4me3, as proposed for other model organisms.

**Supplementary Information:**

The online version contains supplementary material available at 10.1186/s13072-024-00532-y.

## Background

The genus *Fusarium* belongs to the most destructive group of fungal plant pathogens, with two prominent species already ranked amongst the top 10 phytopathogens worldwide [1, 2]. Thus far, approximately 400 genetically diverse *Fusarium* spp. are recognized and separated into 23 species complexes with many more to follow within the next century [3, 4]. In general, fusaria are fungal predators, able to induce a plethora of plant diseases on a myriad of economically important staple crops [5]. Moreover, fusaria own an enormous and yet largely unexplored potential to synthesize a diverse spectrum of small molecular weight compounds also referred to as natural products (NPs) or secondary metabolites (SMs) with often noxious properties [6, 7]. Some of them are produced during plant colonization by the fungus, thereby contaminating precious food and feed sources [8, 9]. Thus, *Fusarium*-induced plant diseases pose unprecedented consequences for the global food and feed security now and in the near future (Fones et al., 2020).

Within the genus *Fusarium*, one of the most well-studied species complexes is the *Fusarium fujikuroi* species complex (FFSC) [10, 11]. Its namesake *Fusarium fujikuroi* is known as a notorious pathogen of rice causing ‘*bakanae*’ (foolish seedling disease) or stunting of rice seedlings [12]. The plant disease is of increasing economic importance due to drastic forfeitures in harvest [13, 14] in all rice-growing countries in the southern hemisphere [15]. Noteworthy, the fungal isolates causing either of the two pathotypes harbor nearly the same set of biosynthetic gene clusters (BGCs) involved in the production of pathogenesis-related NPs but their chemical portfolio differs considerably [11, 12]. Here, gene regulation *via* chromatin structure is a plausible explanation for the observed differences [16, 17].

In every eukaryotic cell, DNA is tightly associated with an octamer of canonical histone proteins to form the basic packaging form of DNA, *i*.*e*., chromatin. Chromatin is a highly dynamic structure providing a platform for the transcriptional machinery. To ensure the timely coordination of gene expression, the chromatin landscape is further shaped by the presence of diverse regulatory elements maintaining the exposure or silencing of the underlying genes [18]. These include histone post-translational modifications (histone PTMs or histone marks) on the histone N-terminal tails, ATP-dependent chromatin-modifying enzyme complexes, or the exchange of canonical histones against non-canonical histone variants [19–22]. Thus, modification of the chromatin structure through the aforementioned mechanisms can either facilitate the opening of the chromatin structure to promote the formation of euchromatin, hence gene transcription, or lead to the compaction of chromatin resulting in inert genomic regions (heterochromatin), consequently gene silencing [18].

The highly conserved histone variant H2A.Z is one of the most well-explored non-canonical histones and has already been intensively studied in model systems such as the yeast *Saccharomyces cerevisiae*, the plant *Arabidopsis thaliana* or the fly *Drosophila melanogaster*. Yet, the role of H2A.Z is still highly controversial and poorly understood. In most tested higher organisms *H2A.Z* is essential, except in *S. cerevisiae*, *Schizosaccharomyces pombe*, and *Neurospora crassa* [23–25] already implying fundamentally pivotal roles for the histone variant H2A.Z. In general, the incorporation of H2A.Z in the nucleosome structure is associated with manifold gene regulatory functions such as gene expression, DNA repair, chromosome stability, epigenetic memory, or cell cycle progression [23, 26–28].

The histone variant H2A.Z is essential in *F. fujikuroi* and *Fusarium graminearum*, however, for the latter lethality is likely rescued through secondary mutations in gene regulatory elements [29]. Interestingly, for the model ascomycete *N. crassa*, NcH2A.Z is dispensable allowing comprehensive studies in this species. Here, incorporation of NcH2A.Z synchronizes fungal circadian rhythm with DNA replication [25], is involved in oxidative stress response [30], is important to maintain normal heterochromatin boundaries [31], and is a prerequisite to establish wild type-like patterns of facultative heterochromatin [32].

Next to this, crosstalk between H2A.Z and some histone marks has been implied. Here, especially a crosstalk between H2A.Z and the two opposing histone marks histone H3 lysine 4 trimethylation (H3K4me3) and histone H3 lysine 27 trimethylation (H3K27me3) involved in active gene transcription and the formation of facultative heterochromatin, respectively, has been shown in metazoan systems and plants alike [23, 33]. Briefly, in *Arabidopsis*, *Oryza sativa*, and mouse embryonic stem cells (mECs), 51%, 77%, and 92%, respectively, of all nucleosomes at promoter regions harboring H2A.Z are also decorated with H3K4me3. In consequence, a positive connection with gene expression is implied [34–36]. However, a negative correlation with facultative heterochromatin decorated with H3K27me3 is observed in others. While co-occupancy of trimethylated H3K27 and H2A.Z actively promotes gene compaction in *A. thaliana* [34], in *N. crassa*, H3K27me3 and H2A.Z are largely mutually exclusive [32].

In the current study, we give a comprehensive overview of the histone variant FfH2A.Z and its role in transcriptional control and explore the possible crosstalk of FfH2A.Z with H3K4me3 and H3K27me3 in the notorious rice pathogen *F. fujikuroi*. By an ‘omics-’oriented approach, we show for the first time the global distribution of FfH2A.Z and the two opposing histone marks H3K4me3 and H3K27me3 in the genus *Fusarium*. Through the incorporation of this data in a nucleosome map, we provide evidence that FfH2A.Z predominantly localizes to the + 1-nucleosome and is largely exclusive with H3K4me3 but not with H3K27me3 unlike described for other model organisms. Global transcriptional data of *F. fujikuroi* and a strain depleted for FfH2A.Z revealed that FfH2A.Z incorporation is highly associated with active gene transcription, especially of genes involved in gene regulatory processes. Most intriguingly, this is also true for genes located within facultative heterochromatin. Lastly, making use of *FfH2A.Z* knock-down and overexpression strains, allowed the first functional characterization of FfH2A.Z in fungal growth and secondary metabolism in a member of the FFSC. Here, our data revealed that the histone variant FfH2A.Z is vital for regular fungal development, including growth and conidiation, but largely dispensable for secondary metabolism in *F. fujikuroi*.

## Result and discussion

### FfH2A.Z is distributed genome-wide and enriched at the + 1-nucleosome near the transcriptional start site

We have previously shown that *FfH2A.Z* is essential in *F. fujikuroi* spp. [29], but its global distribution and function remains enigmatic. To get more insight into the role of FfH2A.Z, we approached chromatin immunoprecipitation followed by sequencing (ChIP-seq) as well as MNase-assisted digestion and subsequent sequencing (MNase-seq) in our reference strain *F. fujikuroi* IMI58289 (FfWT).

For this, FfWT was grown in synthetic liquid ICI supplemented with 6 mM glutamine as the sole nitrogen source for 3 days. For both assays, mycelia were cross-linked before harvest with formaldehyde. Unfortunately, the first ChIP analyses using a commercially available antibody against H2A.Z proved promiscuous and therefore, we proceeded with N- and C-terminal tagging of the native *FfH2A.Z* gene with a 3x-hemagglutinin- (HA) tag. The *FfH2A.Z* ortholog (*FFUJ_01849*) was identified earlier [29] using QuartetS [37]. Multiple sequence alignment with ClustalOmega [38] with already characterized orthologs of H2A.Z revealed 97.9%, 94.4%, and 72.9% amino acid identity with *F. graminearum* (FGSG_01627), *N. crassa* (NCU05347) and *S. cerevisiae* (YOL012C) H2A.Z/Htz1, respectively. Pairwise sequence alignment of FfH2A.Z and FfH2A using EMBOSSNeedle showed an overall amino acid identity of 53.1% and 64.1% similarity. The typical histone fold domain found in the histones H2A, H2B, and H3 as well as the extended C-terminal domain are present in FfH2A.Z (Fig. S1). Interestingly, even though *F. fujikuroi* and *N. crassa* H2A.Z share a sequence homology of 94.4% as well as an identical domain organization, loss of NcH2A.Z is not lethal in this species [30, 32].

**Fig. 1:**
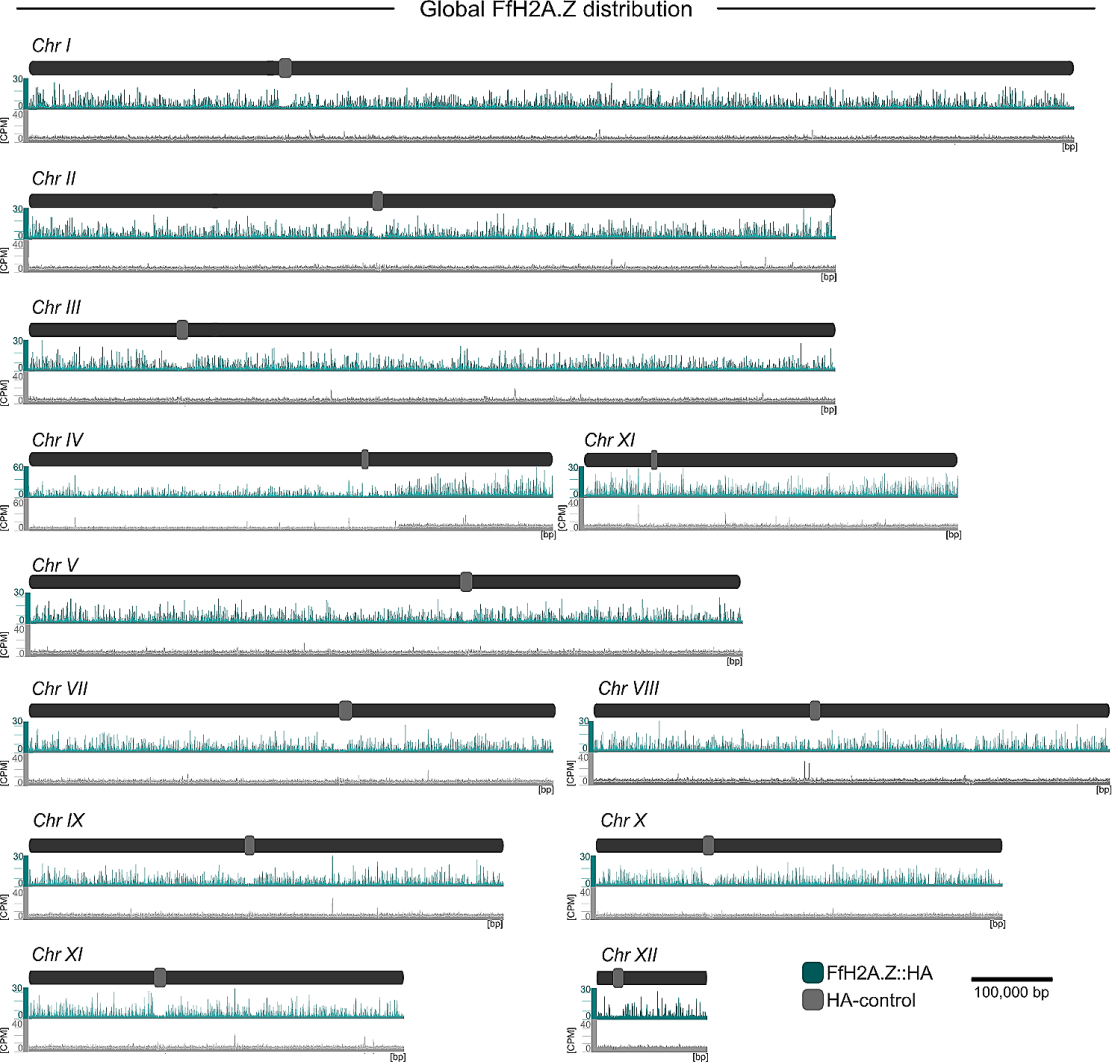
Global distribution of FfH2A.Z in *F. fujikuroi* (FfWT). FfH2A.Z::HA (ChIP-seq) was mapped against the *F. fujikuroi* genome to visualize the overall FfH2A.Z occupancy. Chromosomes are shown in dark gray, while centromeres are depicted in light gray. Genome-wide distribution of FfH2A.Z present in the FfWT strain is depicted in turquoise and absent from the control (light gray). CPM; counts per million reads, bp; base pair

The N- and C-terminal HA-tagged versions of *FfH2A.Z, i*.*e*., HA::*FfH2A.Z* (Fig. S2A) and *FfH2A.Z*::HA (Fig. S3A), respectively, were transformed into the native *FfH2A.Z* locus using a nourseothricin (natR) resistance cassette. Three independent transformants each, *i*.*e*., HA::*FfH2A.Z*_T2, T3, and T4 as well as *FfH2A.Z*::HA_T10, T18, and T21, were gained (Figs. S2B and S3B). Wild type-like *FfH2A.Z* gene expression was confirmed *via* real-time quantitative PCR (RT-qPCR, Figs. S2C and S3C). To verify if tagging of the native *FfH2A.Z* gene influenced fungal development, we assessed radial hyphal growth, asexual reproduction, and SM biosynthesis (Fig. S4A-C). Additionally, for the *FfH2A.Z*::HA strains, a pathogenicity assay was performed (Fig. S4 D). Neither radial hyphal growth, SM production nor pathogenicity were influenced in the *FfH2A.Z*::HA strains, while slightly aberrant growth and SM biosynthesis were observed for the HA::*FfH2A.Z* mutants. Conidiation for both strains was slightly decreased compared to FfWT. Western blot analysis of the N- and C-terminally tagged strains using an anti-HA- and an anti-H2A.Z-specific antibody revealed the successful tagging of FfH2A.Z for HA::*FfH2A.Z*_T2 and T3 as well as for all three independent *FfH2A.Z*::HA mutants (Fig. S4E). Since normal fungal growth was observed for the *FfH2A.Z*::HA strains, we arbitrarily chose *FfH2A.Z*::HA T10 for further analyses. FfWT and the *FfH2A.Z*::HA transformant were grown and harvested as described before and incubated with an anti-HA-specific antibody. Incubation of wild-type mycelia with the HA-antibody served as a negative control to exclude the cross-reactivity of the antibody. For MNase-seq, crosslinked mycelia were treated with MNaseI and mono-nucleosome units were isolated and sent for sequencing. ChIP-seq analysis revealed overall 5,140 +/- 500 (2 biological replicates) FfH2A.Z-specific peaks (counts per million reads (CPM) cutoff > 15), which are evenly distributed over all twelve chromosomes in FfWT (Fig. 1). The cutoff (CPM > 15) was determined by the height of all genome-wide detectable FfH2A.Z peaks falling into a 95% quantile.

**Fig. 2:**
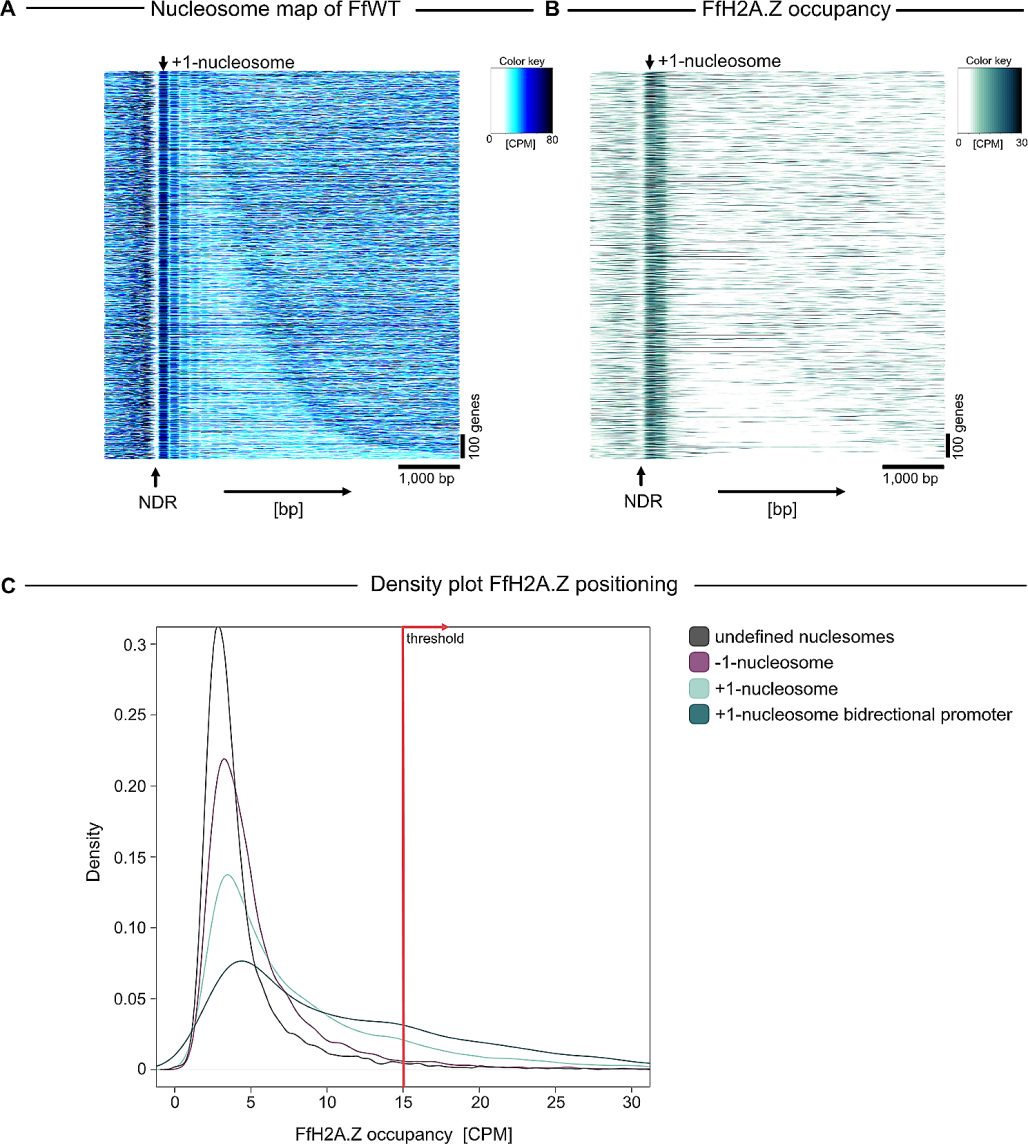
FfH2A.Z occupancy and nucleosome localization in *F. fujikuroi* (FfWT). (**A**) Nucleosome map of all genes in FfWT (1,636 gene regions). Gene regions (1 kilobase pair (kbp) to 5 kbp flanking the + 1-nucleosomes) are sorted by increasing gene length from top to bottom, arrows on the top and on the bottom indicate the position of the + 1-nucleosome and the nucleosome depleted regions, respectively. (**B**) Genes are in the same order as in A, the heatmap shows FfH2A.Z occupancy concerning the + 1-nucleosome. CPM; counts per million reads not log2 scaled, NDR; nucleosome-depleted region, bp; base pair. (**C**) Density plot of FfH2A.Z occupancy over the − 1-, + 1-, + 1-nucleosomes from bidirectional promoters and all other nucleosomes. The threshold was determined based on the height of all genome-wide detectable peaks falling into a 95% quantile. CPM; counts per million reads

From these 3,400 +/-300 peaks ($\sim$60%) are localized within the regions 200 bp upstream to 600 bp downstream of ATG sites. With this, we defined the FfH2A.Z occupation by the maximum of normalized coverage in the regions 200 bp upstream and 600 bp downstream surrounding the ATG-sites of genes (5’ region) being greater than 15 CPM in both replicates (Fig. S5). This resulted in 2,374 promoter regions that are associated with FfH2A.Z incorporation. Next, we used all 2,371 genes harboring FfH2A.Z in their 5’ region to generate nucleosome enrichment maps (Fig. 2A and B) based on the detection of the + 1-nucleosome, *i*.*e*., the first nucleosome downstream the nucleosome-depleted region (NDR) for each gene [39]. From a total of 14,813 annotated genes, for 11,579, the + 1-nucleosome could be detected. This resulted in 1,636 genomic regions where the + 1-nucleosome and FfH2A.Z could be detected. In general, a low nucleosome abundance upstream of annotated genes indicates the transcriptional start site (TSS). This genomic area is also referred to as NDR, a genomic site where DNA is highly accessible for general transcription factors and other chromatin-associated regulators which in turn can recruit RNA polymerase II (RNA Pol II) and initiate transcription of the underlying DNA sequence [40, 41]. The NDR is usually flanked by the − 1- and + 1-nucleosome, defining an upstream and downstream barrier for the transcriptional machinery [42]. In general, for *F. fujikuroi*, nucleosome units are prevalent at the + 1- and to a slightly lesser extent at the + 2- and + 3-nucleosome, as well as at the − 1-nucleosome. This is followed by nucleosome depletion over the gene body **(**Fig. 2A). Aligning the data from the nucleosome map to the FfH2A.Z-specific peaks from the ChIP-seq analysis, clearly showed that FfH2A.Z is present at the + 1- and + 2-nucleosomes but lacking from the − 1-nucleosomes as well as from gene bodies (Fig. 2B). This pattern is seemingly conserved also in other organisms, *i*.*e*., in *S. cerevisiae*, *D. melanogaster*, and *A. thaliana*, where H2A.Z consistently occupies + 1-nucleosome at distinct genetic loci in the direction of transcription [43–45]. For the parasite *Plasmodium falciparum*, PfH2A.Z shows enrichment in euchromatic intergenic regions, *i*.*e*., in the TSS, and spans 6–8 nucleosome units [46, 47]. Next, examining FfH2A.Z incorporation in nucleosome units in more detail, it is evident that FfH2A.Z is almost exclusively incorporated at the + 1-nucleosome. In case where FfH2A.Z is also incorporated at the − 1-nucleosome, it seems as if these nucleosomes function at the same time as the + 1-nucleosomes for another gene, *i*.*e*., bidirectional promoter (Fig. 2C), which is also true for *S. cerevisiae* [45]. However, analyses in human cells showed that both nucleosomes flanking the TSS are indeed occupied by H2A.Z [48].

To conclude, the histone variant FfH2A.Z is predominantly incorporated non-randomly at the + 1-nucleosome on a genome-wide level in *F. fujikuroi*. Here, a larger fraction of FfH2A.Z is incorporated at the + 1-nucleosome of bidirectional promoters, which may serve simultaneously as a -1-nucleosome for the opposite gene.

### FfH2A.Z is enriched at the 5’ region of actively transcribed genes in *F. fujikuroi*

The histone variant H2A.Z assumes pivotal roles in shaping the transcriptional landscape as already reported for several organisms [21, 26]. As we were curious to study the role of FfH2A.Z in transcriptional regulation also in *F. fujikuroi* in more detail, we first approached down-regulation of *FfH2A.Z* gene expression *via* the dosage-dependent tetracycline-responsive knock-down system TetOff (TetOff::*FfH2A.Z*) [49] in combination with an in situ constitutive overexpression of *FfH2A.Z* (OE::*FfH2A.Z*) in FfWT.

**Fig. 3:**
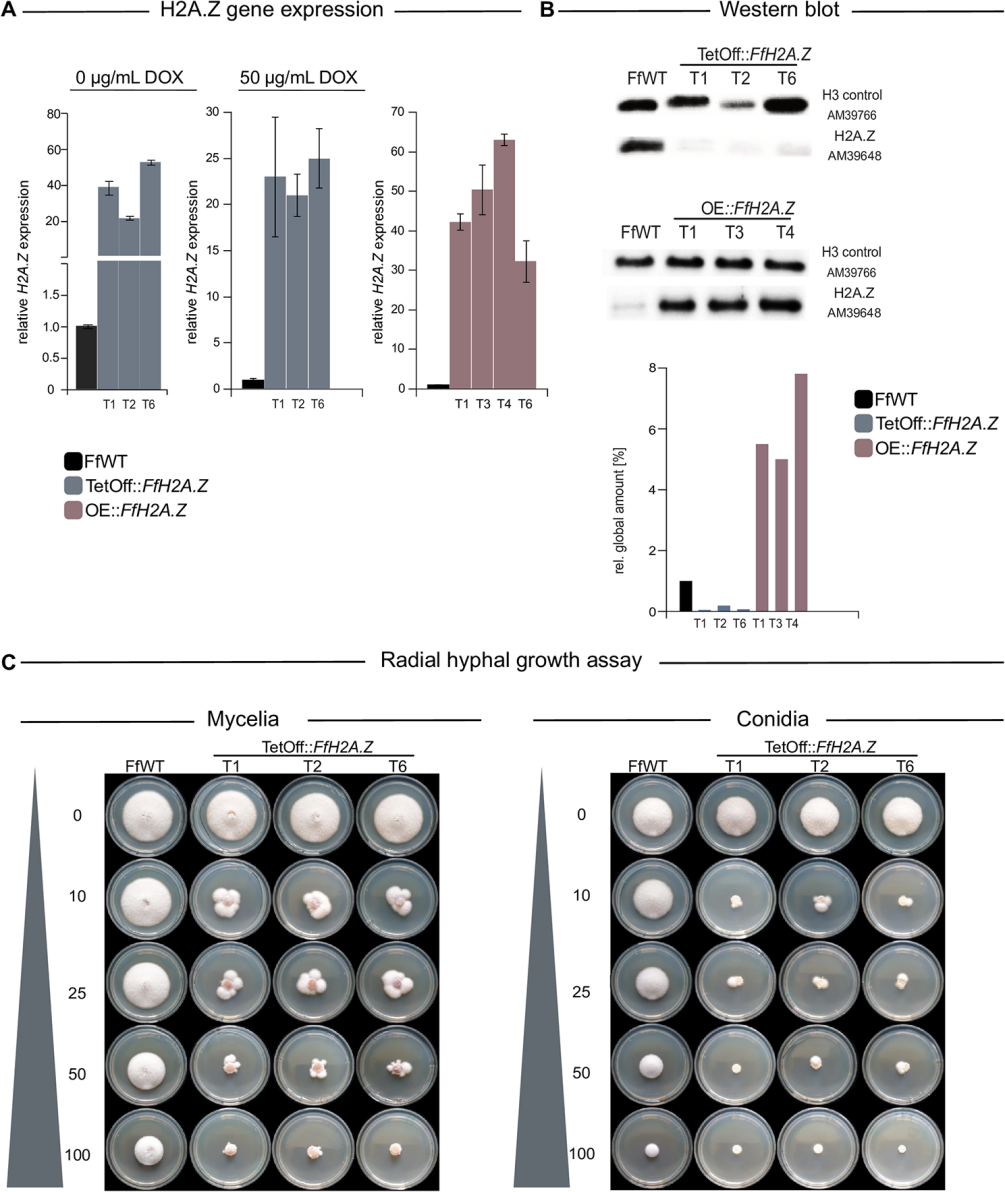
Verification and characterization of OE::*FfH2A.Z* and TetOff::*FfH2A.Z* strains in *Fusarium fujikuroi* (FfWT). (**A**) Expressional analysis of the FfH2A.Z mutant strains OE::*FfH2A.Z* and TetOff::*FfH2A.Z via* RT-qPCR. For the FfH2A.Z depletion strain, *FfH2A.Z* expression was measured upon cultivation on CM supplemented with (50 µg/mL) and without (0 µg/mL) the inducing reagent doxycycline hyclate (DOX). The *FfH2A.Z* overexpression strain was cultivated on CM at 30 °C for 3 days in the dark. Gene expression of FfWT was arbitrarily set to 1. (**B**) Western blot analysis of FfWT, *FfH2A.Z* knock-down and overexpression mutant strains. Proteins were probed with an anti-H2A.Z-specific and an anti-H3 C-terminus-specific antibody. For quantification, a densitometric analysis was performed where FfWT was arbitrarily set to 1. (**C**) Assessment of radial hyphal growth of the inducible TetOff::*FfH2A.Z* strains and FfWT on CM using different concentrations of the inducing agent DOX. The medium was inoculated either with a mycelial plug (left panel) or 1,000 conidia each (right panel) and incubated for 4 days post inoculation at 30 °C in the dark. Experiments were performed in biological triplicates

Strain generation of the knock-down and overexpression of *FfH2A.Z* was performed *via* homologous integration of the respective constructs into the native *FfH2A.Z* locus using a hygromycin resistance (hygR) cassette for the positive selection of transformants (Figs. S6A and S7A). Three independent *FfH2A.Z* knock-down transformants were gained, *i*.*e*., TetOff::*FfH2A.Z_*T1, T2, and T6 (Fig. S6B), while several transformants harboring the *FfH2A.Z* overexpression construct were obtained and arbitrarily three of them (OE::*FfH2A.Z*_T1, T3 and T4, Fig. S7B) were chosen for further analysis. Successful knock-down as well as overexpression of *FfH2A.Z* was verified *via* RT-qPCR and western blot analysis (Fig. 3A and B) using a commercially available anti-H2A.Z-specific antibody. To our surprise, *FfH2A.Z* expression in the knock-down strains under the addition of doxycycline hyclate (DOX) exceeded the expression of FfWT. However, western blot analysis clearly showed a nearly complete loss of FfH2A.Z on protein level under the same culture conditions. Later RNA-sequencing (RNA-seq) analysis of two independent knock-down mutants revealed wrong splicing patterns for *FfH2A.Z* transcripts, thus explaining increased transcription levels but lack of FfH2A.Z on protein level (Fig. S8). All transformants displayed a similar phenotype.

Silencing *via* the TetOff system is dosage-dependent as shown earlier [49]. To determine the ideal DOX supplementation concentration, fungal growth on solid CM supplemented with different concentrations of DOX (0-100 µg/mL) was performed. Plates were point-inoculated either with freshly grown fungal mycelia or 10 µL of a 10^5^ conidia/mL suspension of FfWT or TetOff::*FfH2A.Z*, respectively. Plates were incubated at 30 °C for 4 days. As depicted in Fig. 3C, reduced hyphal growth was observed for the knock-down strains already at 10 µg/mL DOX on plates inoculated either with mycelia or conidia. However, wild type-like “segmented” mycelia were observed next to crippled mycelia under the supplementation of nearly all tested DOX concentrations. The most stable growth of the mutant strains was observed under the addition of 100 µg/mL DOX but growth of FfWT was impeded drastically as well. To balance fungal growth and sufficient *FfH2A.Z* silencing, 50 µg/mL DOX was used for the induction of the TetOff::*FfH2A.Z* strains from now on.

**Fig. 4:**
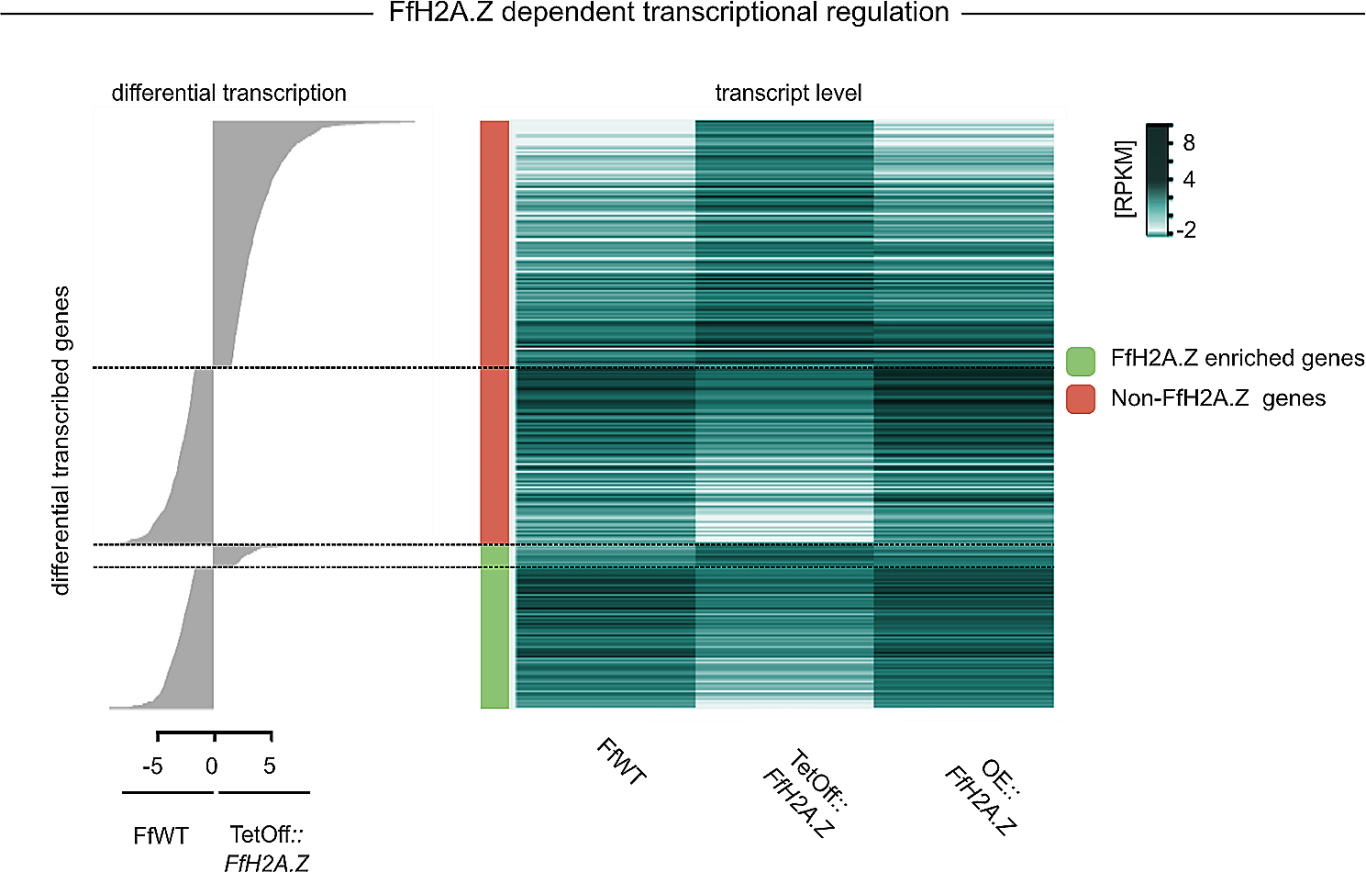
Global correlation between gene transcription and FfH2A.Z incorporation. The left-hand side (gray bars) shows barplot differential transcription of genes ordered by FfH2A.Z presence in the 5’ region and the level of differential transcription. The middle bar (red) indicates genes with no FfH2A.Z in their 5’ region, green indicates genes decorated with FfH2A.Z. The right-hand side shows a heatmap of transcription levels (RPKM; reads per kilobase pairs per million, normalized by log2) in the *Fusarium fujikuroi* wild type-strain (FfWT), the FfH2A.Z depletion strain (TetOff::*FfH2A.Z*), as well as the *FfH2A.Z* overexpression strain (OE::*FfH2A.Z*).

Next, we performed RNA-seq with OE::*FfH2A.Z*, the TetOff::*FfH2A.Z*, and the respective wild-type strain using mycelia cultivated under the same conditions as used for ChIP- and MNase-Seq. Using a threshold of ≥ ± 1 for differential expression (log2) and a *p*-value < 0.01, 1,225 genes were upregulated and 1,455 genes were downregulated in the TetOff::*FfH2A.Z* mutant compared to FfWT, respectively (Table S1). Overall, 18% of the genome is deregulated, indicating neither an activating nor a repressing regulatory function of FfH2A.Z on a global scale. For the OE::*FfH2A.Z* strains, only 418 genes were up- and 180 downregulated, respectively (Table S1). Examining all genes that harbor FfH2A.Z in their 5’ region in more detail, we found that only 110 genes were up-, while 642 genes were downregulated by FfH2A.Z depletion. Thus, indicating that FfH2A.Z positively correlates with gene expression when incorporated near the TSS in *F. fujikuroi *(Fig. 4). These findings are similar as already shown for the ascomycete *N. crassa* [32], human CD4^+^ T cells [48, 50], and as indicated for *Tetrahymena thermophila* [51] where a positive correlation between H2A.Z incorporation and gene transcription was reported. This, however, stands in marked contrast to what has been described for other organisms such as *S. cerevisiae* [45], *A. thaliana* [36], *D. melanogaster* [52], and mammalian cells [53, 54], where a more dual role is ascribed to H2A.Z. Briefly, in *S. cerevisiae* ScH2A.Z marks the TSS of both silent and actively transcribed genes [45, 55] and the presence of ScH2A.Z at poorly transcribed genomic loci or even at heterochromatic regions [56, 57], where it is attributing to gene silencing, is evident. However, a recent study has shown that ScH2A.Z occupancy could not be unequivocally connected to either gene expression or gene silencing [45]. It appears that at this point, merely correlating H2A.Z occupancy to the transcriptional output is more complex than initially anticipated. However, it is noteworthy to mention that acetylation of H2A.Z (H2A.Zac) has been linked to positive gene transcription in several cell lines from chickens [58] to mammals [59, 60]. If this is also true for *F. fujikuroi* remains elusive at this point and is subject to further research. Hence, the regulatory role of H2A.Z in gene transcription remains somehow controversial depending on the organism.

To study the function of genes affected by FfH2A.Z depletion in more detail, a Gene Ontology – Biological Processes (GO-BP)-based enrichment analysis of different subsets of genes was performed. Analysis of the genes occupied by FfH2A.Z revealed that 738 from 2,371 genes harboring FfH2A.Z are annotated by GO-BP and a total of 507 thereof are associated with: cellular macromolecule biosynthetic process (294), cellular macromolecule metabolic process (380), cellular nitrogen compound metabolic process (442), gene expression (361), nucleic acid metabolic process (362), regulation of biosynthetic process (214), regulation of nitrogen compound metabolic process (217) and regulation of transcription by RNA polymerase II (181) (Table S2). These results not only indicate that FfH2A.Z is crucial for general gene regulatory processes such as transcriptional control in *F. fujikuroi*, but also serve as a putative explanation for the observed secondary effects on the transcriptional levels in genes most likely not enriched in FfH2A.Z (Fig. 4). Next, to support this assumption, GO-BP annotation on both subsets of genes, *i*.*e*., positively or negatively affected by the FfH2A.Z depletion, was performed. Here, genes that are down-regulated in the TetOff::*FfH2A.Z* mutant strain indeed showed strong associations with amino acid activation (9), biosynthetic process (215), cellular biosynthetic process (199), cellular macromolecule biosynthetic process (147), cellular macromolecule metabolic process (178), cellular nitrogen compound biosynthetic process (161), gene expression (165), regulation of cellular biosynthetic process (97), regulation of metabolic process (103) and RNA metabolic process (140), which is in line with the results obtained in the GO-BP analysis performed on genes enriched in FfH2A.Z (Table S3). Hence, this further supports the assumption, that FfH2A.Z comprises vital roles in guarding the overall transcriptional balance in *F. fujikuroi*.

Lastly, when probing the gene set, which shows upregulation in the TetOff::*FfH2A.Z* mutant strain, GO-BP analysis revealed an enrichment in cellular response to DNA damage stimulus (17), chromosome organization (21), chromosome segregation (11), DNA metabolic process (23), establishment of chromosome localization (3), mitotic metaphase plate congression (3), positive regulation of chromosome segregation (3), response to mitotic spindle checkpoint signaling (2), tyrosine catabolic process indicating important. In general, H2A.Z is not only described to be involved in transcriptional control but also governs other fundamental roles, such as DNA repair and genome stability [61–63], which is also indicated by this analysis. Underrepresentation of FfH2A.Z leads to the upregulation of genes involved in chromosome stability and DNA repair. Interestingly, studies in *S. cerevisiae* revealed that loss of ScH2A.Z resulted in genetic instability caused by the accumulation of recombinogenic DNA damage through the non-coordinated action of the ATP-dependent remodeling complex SWR1 [64] and in consequence promoted genetic instability. It is tempting to hypothesize that genomic instability is the cause for the observed lethality upon loss of FfH2A.Z in *F. fujikuroi* [29]. However, this assumption needs further proof.

### FfH2A.Z induces gene transcription regardless of the chromatin state

In plants and higher eukaryotes, a correlation between histone marks, the histone variant H2A.Z, and gene transcription is evident [23, 33]. In detail, H2A.Z colocalization with histone marks at H3 and their impact on the transcriptional output are already well described for histone H3 lysine 4 trimethylation (H3K4me3) as well as histone H3 lysine 27 trimethylation (H3K27me3) in organisms such as the model plant *A. thaliana* [36], mESCs [34] or the ascomycete fungus *N. crassa* [32]. In general, H3K4me3 and H3K27me3 are of uttermost interest, since both marks are considered to act antagonistically regarding gene transcription. H3K4me3 mostly correlates with active gene transcription and is prominent in loosely packed chromatin (euchromatin), while H3K27me3 is deposited at transcriptionally inert regions with high nucleosome occupancy (facultative heterochromatin) [18, 20].

**Fig. 5:**
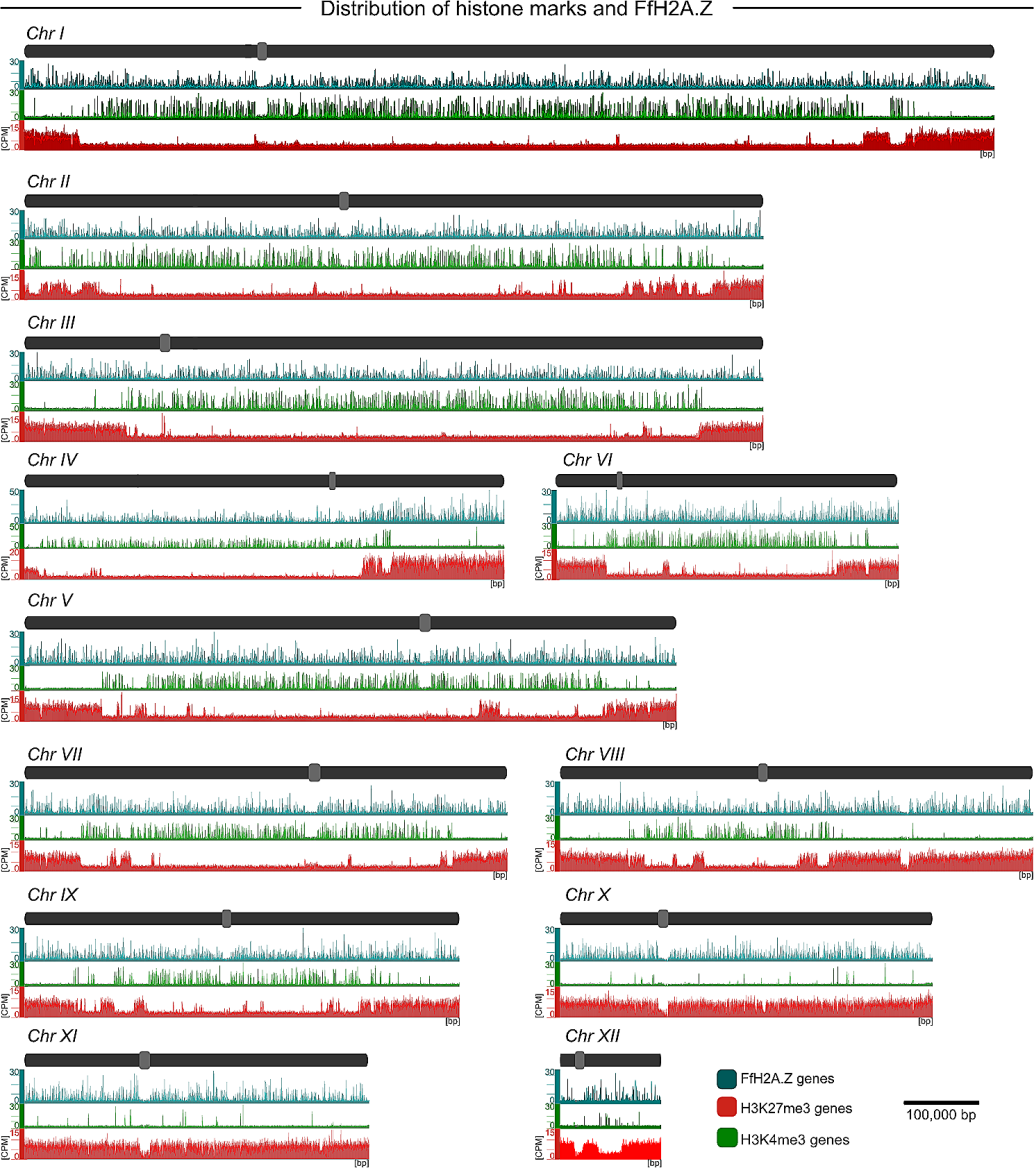
Global distribution of H3K4me3, H3K27me3, and FfH2A.Z in *F. fujikuroi* (FfWT). All twelve FfWT chromosomes and their overall FfH2A.Z, H3K4me3, and H3K27me3 distribution. Chromosomes are shown in dark gray, while centromeres are depicted in light gray. FfH2A.Z occupancy is shown in turquoise, while decoration with H3K4me3 and H3K27me3 is shown in green and red, respectively. CPM; counts per million reads, bp; base pair

To test whether a similar co-localization pattern of H3K4me3 or H3K27me3 with the histone variant FfH2A.Z also exists in *F. fujikuroi*, FfWT mycelium was incubated with an anti-H3K4me3- and an anti-H3K27me3-specific antibody, respectively, followed by ChIP-seq. Obtained results show that overall 4,997 genes are enriched for H3K27me3 (max coverage upstream 100 bp downstream 1000 bp from ATG > 15 CPM), while 2,008 genes are decorated with H3K4me3 (max. coverage upstream 200 bp, downstream 600 bp from ATG > 20 CPM). In *F. fujikuroi*, H3K4me3, and H3K27me3 are largely exclusive (only 6 genes harbor both histone marks) on a genome-wide level (Fig. 5). Similar findings have been observed in *Leptospheria maculans, Zymoseptoria tritici*, *Podospora anserina*, and *F. graminearum* where both histone marks are either mutually or largely exclusive [65–68].

**Fig. 6:**
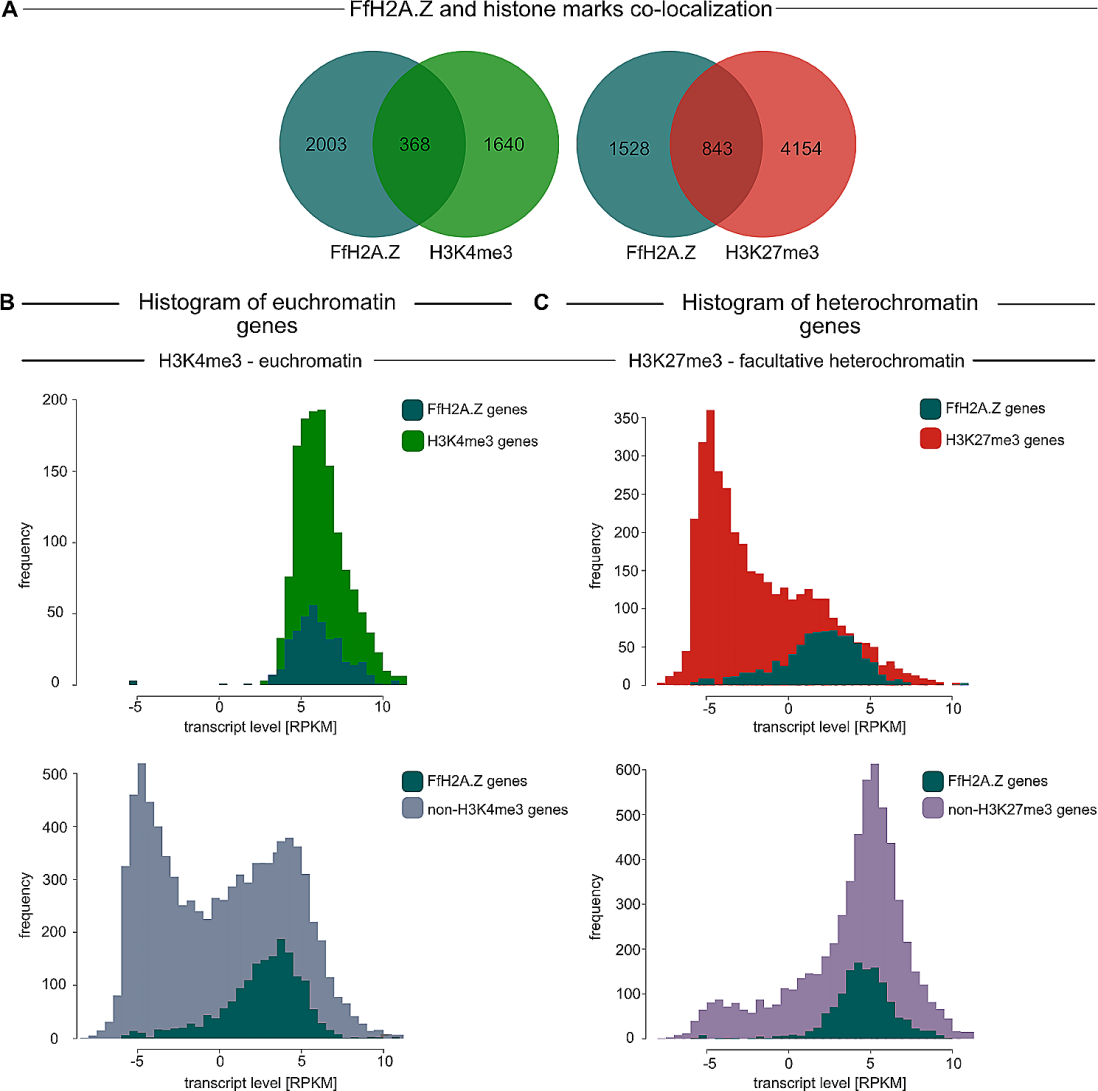
Connection between histone mark distribution, FfH2A.Z, and their correlation with transcription in *F. fujikuroi *(FfWT). (**A**) Venn diagram of FfH2A.Z-marked genes versus H3K4me3- and H3K27me3-labeled genes, respectively. (**B**) Histogram of transcription levels in H3K4me3-/non-H3K4me3-enriched genes with and without the presence of the histone variant FfH2A.Z. (**C**) Histogram of transcriptional output in H3K27me3-/non-H3K27me3-enriched genes with and without FfH2A.Z. RPKM, reads per million per 1 kilobase (kb) normalized by log2

Comparison with the above described FfH2A.Z distribution data reveals that approximately 36% or 16% of all FfH2A.Z-containing nucleosomes are incorporated at genomic loci decorated with H3K27me3 or H3K4me3, respectively (Fig. 6A). If we now compare all genes decorated with either H3K27me3 (3688 genes) or H3K4me3 (1407 genes), which are not marked by FfH2A.Z (10,909 genes), we find 34% and 13% of facultative heterochromatin and trimethylated H3K4, respectively, in non-FfH2A.Z-marked genomic regions. These results already indicate that the presence of both, trimethylated H3K27 and H3K4 is largely independent of FfH2A.Z incorporation. Interestingly, for *A. thaliana*, *O. sativa*, *P. falciparum*, and mESCs a much higher co-occupancy rate between H3K4me3 and H2A.Z ($\sim$50 − 92%) was observed, compared to H3K27me3 ($\sim$20%) [34–36, 46, 47]. For the ascomycete *N. crassa*, the presence of NcH2A.Z is a prerequisite to establish H3K27me3 even if the histone variant and facultative heterochromatin are largely exclusive [32].

Correlating the transcriptional output to the H3K27me3- and H3K4me3-enriched regions shows, as expected, that H3K27me3 localizes to transcriptionally inert regions, while H3K4me3 is enriched in regions that are predominantly transcriptionally active (Table S1). This is in line with data obtained for *F. fujikuroi* [69, 70] and various other ascomycetes. Next, we investigated the relationship between H3K4me3/H3K27me3, FfH2A.Z and their role in transcriptional control and plotted the frequency of genes with and without the presence of FfH2A.Z against the transcript levels (Fig. 6B and C). As anticipated, euchromatic genes labeled with H3K4me3 exhibit high transcription levels independent of FfH2A.Z incorporation. If we now look at all genes harboring both FfH2A.Z and H3K4me3 (368 genes), 85 are upregulated, while only 2 genes are downregulated in FfWT compared to the *FfH2A.Z* knock-down mutant. Thus, we could not determine a clear connection between the presence of FfH2A.Z and the trimethylation of H3K4 (Fig. 6B, upper left panel). However, a clear connection between H2A.Z and H3K4me3 in regard to transcriptional control is shown in certain cases. Here, for the parasite *P. falciparum* and the rice plant *O. sativa*, the presence of H2A.Z in euchromatic regions marked by H3K4me3 is associated with gene expression [35, 46]. In ESCs, H2A.Z is colocalized with H3K4me3 at active regulatory regions, which are readily transcribed [34]. Next to this, in *S. cerevisiae* both ScH2A.Z and H3K4me3 are vital to antagonize telomeric heterochromatin spread by the Sir2-4 complex and thus preventing gene silencing [71]. In *F. fujikuroi*, such a correlation is not as clear as initially anticipated. Here, unlike observations in other eukaryotes, only 16% of all FfH2A.Z is found in H3K4me3-marked genomic regions. Next, when all genes not decorated with H3K4me3 (remaining euchromatic and heterochromatic) are aligned to the presence of FfH2A.Z, it is evident that the large majority of genes harboring FfH2A.Z in their 5’ region is transcribed (Fig. 6B, lower left panel), thereby supporting our assumption that incorporation of FfH2A.Z induces gene transcription in *F. fujikuroi*.

Facultative heterochromatin is defined by the presence of trimethylation at H3K27 and therefore genes solely primed by this mark predominantly stay silent (this study, [69]). Most interestingly, if H3K27me3 colocalizes with FfH2A.Z, a large fraction of genes is transcribed (Fig. 6C, upper right panel). This observation is opposed to what has been described in other eukaryotes thus far. In the model plant *A. thaliana* and *O. sativa*, the presence of H2A.Z in facultative heterochromatic genomic regions, especially at enhancers, facilitates gene silencing and chromatin compaction [35, 36]. In ECSs, H2A.Z and Polycomb Repressive Complex 2 (PRC2) components, which are essential for establishing H3K27me3, accumulate at developmentally silent genes [72, 73] to promote chromatin compaction. In filamentous fungi, the main mechanisms acting on facultative heterochromatin to make it accessible for the transcriptional machinery remain elusive, but it is tempting to speculate that incorporation of FfH2A.Z can counteract gene silencing by H3K27me3 and promote gene transcription. Next, if all genes, which are not marked as facultative heterochromatin are aligned to the transcriptional data, a large fraction of these are transcribed and if these genes colocalize with the histone variant FfH2A.Z, nearly all of them are readily expressed (Fig. 6C, lower right panel).

Intrigued by these findings, we wanted to explore if FfH2A.Z- or H3K4me3- and H3K27me3-decorated genes show higher evolutionary conservation with respect to the overall genomic genes. Therefore, we used the functional NCBI-BLASTP and the whole proteome of *F. fujikuroi* to search for homologous genes in the refseq_protein (NCBI) database. We are aware of the bias within this database towards genes that are more frequently transcribed since these are better annotated compared to rarely expressed genes. However, our data indicates that H3K27me3-decorated genes are the least conserved, and H3K4me3-decorated genes are the most conserved (Table S4). Interestingly, we could not detect a better conservation score in FfH2A.Z-decorated genes that also reside in facultative heterochromatic regions. As expected, we, could determine that genes, that are decorated with histone marks associated with active gene transcription (H3K4me3 and FfH2A.Z) are evolutionary more conserved than genes decorated with a repressing histone mark (H3K27me3). This means that genes marked by FfH2A.Z and H3K27me3 are not higher conserved than genes solely primed by facultative heterochromatin.

To sum up, these results support the prior observations, *i*.*e*., when FfH2A.Z is coordinately incorporated in the 5’ region of certain genes, these are predominantly poised for gene transcription. Thus, the eviction of FfH2A and incorporation of FfH2A.Z, especially at genes involved in gene regulatory processes, promotes gene transcription in *F. fujikuroi*. Even more surprisingly, this phenomenon occurs regardless of the chromatin state, *i*.*e*., facultative heterochromatin or euchromatin, marked by H3K27me3 or H3K4me3, respectively. It is noteworthy to mention, that we only used one culture condition for our experiments, thus genes marked with FfH2A.Z could be prone to differential transcription, depending on the growth conditions and the present histone marks. Future experiments in different culture conditions will shed more light onto this matter.

Additionally, we show that genes marked with FfH2A.Z and H3K4me3 are higher conserved than genes marked with FfH2A.Z and H3K27me3, indicating that incorporation of FfH2A.Z does not selectively incorporate at genomic loci which are conserved to a higher degree.

### Depletion of FfH2A.Z promotes gene silencing in facultative heterochromatic regions

As we were now curious to determine H3K4me3 and H3K27me3 patterns and their role in the transcriptional output in the FfH2A.Z-depleted strain, TetOff::*FfH2A.Z* (synthetic ICI, 6 mM glutamine, 50 µg/mL DOX) was incubated with a H3K4me3- and a H3K27me3-specific antibody, respectively, followed by ChIP-seq.

**Fig. 7:**
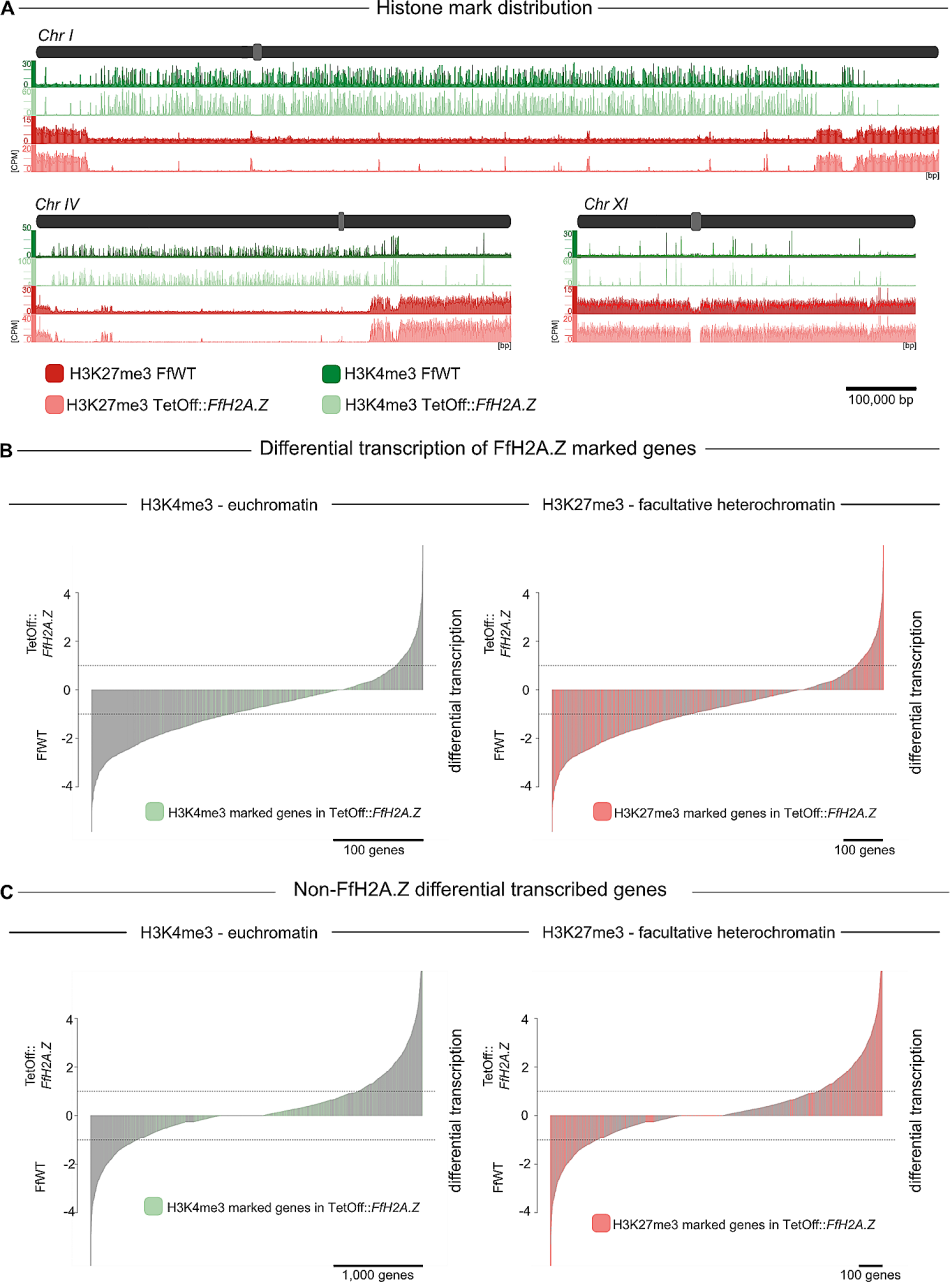
Global distribution of H3K27me3 and H3K4me3 and their role in gene transcription in TetOff::*FfH2A.Z*. (**A**) Global view on *F. fujikuroi* (FfWT) and TetOff::*FfH2A.Z* chromosomes I, IV, and XI and respective distribution of H3K4me3 and H3K27me3. Chromosomes I, IV, and XI are shown in dark gray, while centromeres are depicted in light gray. Genome-wide distribution of H3K4me3 is shown in green for FfWT and in light green for the FfH2A.Z knock-down strain. The global view of H3K27me3 in FfWT is depicted in red, while the mutant strain is shown in light red. CPM; counts per million reads, bp; base pair. (**B**) Visualization of FfH2A.Z-enriched and differentially transcribed genes (TetOff::*FfH2A.Z* – FfWT). In the left panel, differentially transcribed genes are shown in gray, while light-green markings indicate the presence of H3K4me3. The right panel represents FfH2A.Z-enriched genes (gray) and their differential transcription compared to FfWT. Genes decorated with facultative heterochromatin are marked in light red. (**C**) Depiction of remaining non-FfH2A.Z genes in regard to differential transcription. Differently transcribed genes are shown in gray (left and right panel), while H3K4me3-decorated genes are depicted in light green, and H3K27me3-enriched genes are shown in light red. The dashed lines determine the confidence interval of ≥ ± 1

Comparing global patterns of both, H3K4me3 and H3K27me3, of the *FfH2A.Z* knock-down strain with FfWT, revealed that no major loss, gain, or re-distribution of both histone marks occurred in the near absence of FfH2A.Z in the mutant strain (Fig. 7A and S9). However, a subtle global gain of H3K4me3 peaks was observed, which is most likely attributable to the better signal-to-noise ratio of the knock-down strain compared to the wild type. The reason for this could be a better shearing efficiency of the aberrant mycelium of the knock-down strain. As both histone marks are overall distributed in a wild type-like manner in the TetOff::*FfH2A.Z* strain, it is likely that the presence of FfH2A.Z is not linked to the establishment of facultative heterochromatin (H3K27me3) or the euchromatic histone mark H3K4me3 in *F. fujikuroi*. Unlike *F. fujikuroi*, in *N. crassa*, the decoration with H3K27me3 is linked to the incorporation of NcH2A.Z. Here, the removal of NcH2A.Z, which is otherwise present at the promoter of the PRC2 member gene *eed*, resulted in subdued *eed* expression, which in consequence resulted in reduced levels of H3K27me2/3 [32]. This is similar to observations for ESCs, where H2A.Z is crucial for establishing normal patterns of H3K27me3 at bivalent domains [74]. Next to this, an earlier study in ESCs revealed that knock-down of *H2A.Z* led to drastically reduced H3K27me3 levels, especially at enhancers located in facultative heterochromatic regions, while only subtle changes for trimethylation of H3K4 were observed [34].

Now, to investigate the relationship between the two prominent histone marks, *i*.*e*., H3K4me3 and H3K27me3, and FfH2A.Z in more detail, the transcriptional output was aligned to the ChIP-seq data obtained from the *FfH2A.Z* knock-down mutant (Fig. 7B and C). As mentioned before, roughly 13% of all genes harboring FfH2A.Z colocalize with H3K4me3. Not surprisingly, only a few genes trimethylated at H3K4 are de-regulated (positively and negatively) on the transcriptional level, after *FfH2A.Z* knock-down (Fig. 7B, upper left panel). The same is true for genes indirectly affected by the underrepresentation of FfH2A.Z (Fig. 7B, lower left panel), *i*.*e*., only a fraction of differentially transcribed genes are decorated with H3K4me3. This is rather surprising and unlike what has been proposed for other eukaryotic systems where a co-dependency of H2A.Z, H3K4me3, and the transcriptional output has been reported. In contrast, our data indicates that FfH2A.Z acts independently of H3K4me3 on the transcriptional level. It is possible that both regulatory elements are positively correlated with transcriptional output, albeit being targeted to different sets of genes. Still, approximately 1/3 of all genes priorly enriched with FfH2A.Z remain covered with H3K27me3. Intuitively, through the depletion of FfH2A.Z, most of these genes turn transcriptionally silent (Fig. 7B, upper right panel). It is tempting to speculate that the incorporation of FfH2A.Z at the + 1-nucleosome causes increased accessibility at the NDR, despite the heterochromatic structure. Hence, genes silenced through the action of facultative heterochromatin get poised for gene transcription by the incorporation of FfH2A.Z in their 5’ region. As a consequence, the depletion of FfH2A.Z leads to the silencing of these genes, which are now inaccessible to the transcriptional machinery. However, the lack of a Δ*ffH2A.Z* deletion strain or ChIP-seq data on the globally remaining FfH2A.Z levels in the *FfH2A.Z* knock-down mutant, makes it hard to connect the FfH2A.Z-mediated transcriptional output to H3K27me3-marked regions. Thus, these assumptions must be proven with further experiments. Yet, a small fraction of genes, despite the presence of H3K27me3, gets transcribed (Fig. 7B, upper right panel). The reason for this is currently unclear. The same is true for genes, which are indirectly impacted on the transcriptional level by *FfH2A.Z* knock-down (Fig. 7C, lower right panel). Here, non-FfH2A.Z genes are up- and down-regulated regardless of the presence of H3K27me3. A possible explanation for this could be that FfH2A.Z is predominantly incorporated near genes associated with gene regulatory processes, hence, lack of FfH2A.Z may cause other genetic elements to induce gene transcription in regions that are otherwise transcriptionally inert.

To conclude, depletion of the histone variant FfH2A.Z does not cause major changes in patterns of the two opposing histone marks, i.e., trimethylation of H3K4 and H3K27, associated with gene transcription and silencing, respectively. Unlike as proposed for several other eukaryotic organisms, our data point towards a largely exclusive role of the histone variant FfH2A.Z and H3K4me3 in transcriptional regulation. Next to this, the presence of FfH2A.Z in facultative heterochromatic regions facilitates gene transcription, whereas the global reduction of FfH2A.Z levels largely results in gene silencing in these regions.

### The incorporation of FfH2A.Z supports the + 1-nucleosome positioning in H3K27me3-marked regions

**Fig. 8:**
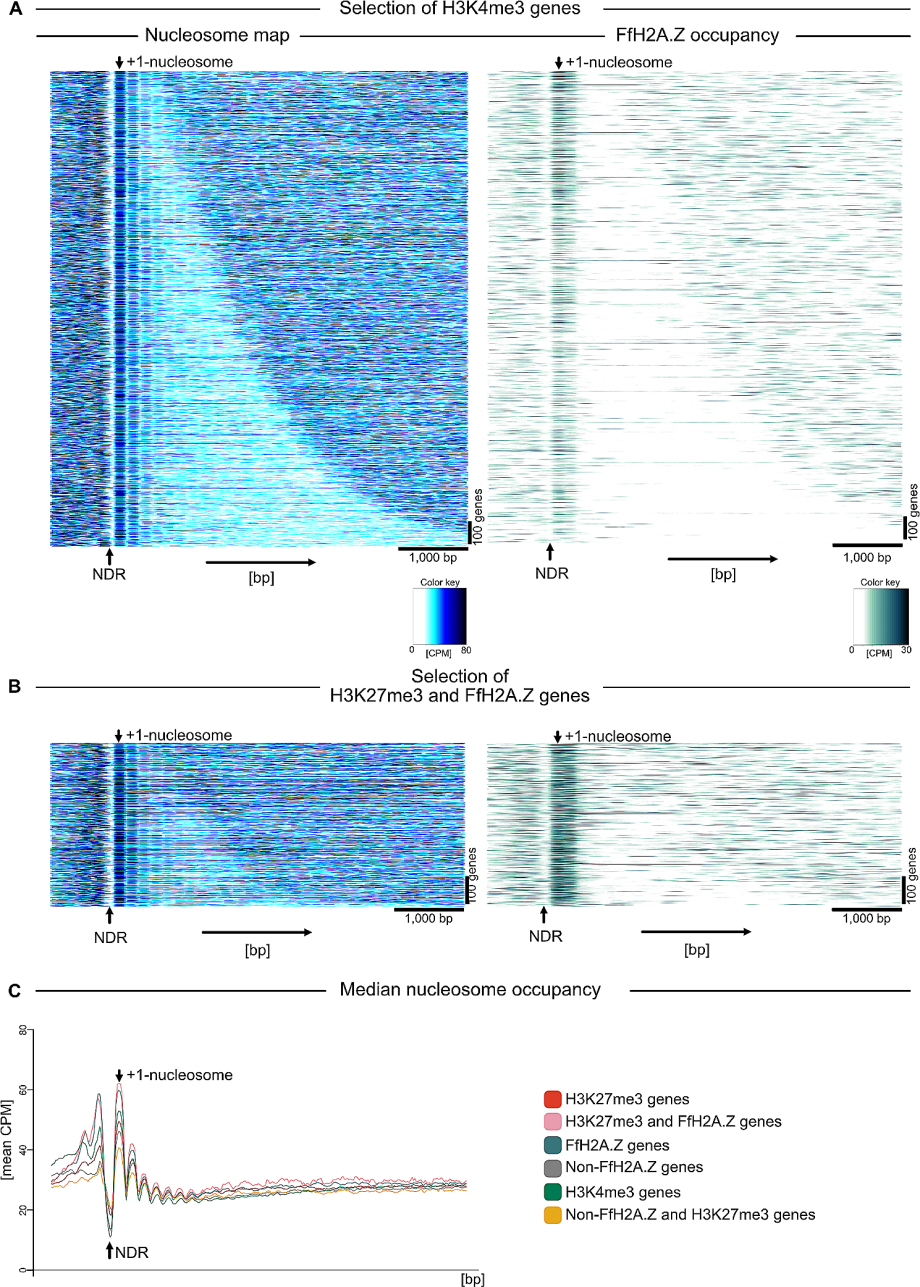
+ 1-nucleosome positioning in H3K4me3- and H3K27me3-marked regions in correlation to FfH2A.Z occupancy. (**A**) Nucleosome map of all H3K4me3-marked genes (left panel) and FfH2A.Z-enriched genes in this genomic region. The right panel shows the overall FfH2A.Z occupancy with respect to the + 1-nucleosome. CPM; counts per million reads not log2 scaled, bp; base pair, NDR; nucleosome-depleted region. (**B**) The left panel shows a nucleosome map of genes enriched with FfH2A.Z and covered with H3K27me3, while the right panel depicts the overall FfH2A.Z occupancy with respect to the + 1-nucleosome. Genes are sorted by increasing gene length from top to bottom, arrows top and bottom indicate the position of the + 1-nucleosome and the nucleosome-depleted regions. (**C**) The median nucleosome occupancy (mean CPM, mean counts per million reads) plot shows for each specified subset of genes the median per base pair (binned by 21 bp) thereby summarizing the specific heatmaps of nucleosome occupancies. NDR; nucleosome-depleted region, bp; base pair

As we now were curious to investigate, whether FfH2A.Z incorporation leads to a stronger + 1-nucleosome positioning in H3K4me3- as well as H3K27me3-marked regions, we generated nucleosome maps of all H3K4me3-marked genes and genes covered with H3K27me3 harboring FfH2A.Z in their 5’ region. Here, all genes trimethylated for H3K4 show a relatively robust + 1-nucleosome positioning thus, accessibility of the NDR, for the transcriptional machinery (Fig. 8A, left panel). This is in accordance with the prior findings, that all H3K4me3-marked genes are transcribed (Fig. 6B, upper left panel). However, our data show that the + 1-nucleosome positioning in these genomic areas is largely independent of the presence of the histone variant FfH2A.Z (Fig. 8A, right panel). Alignment of FfH2A.Z to the H3K4me3-nucleosome map, revealed no strong correlation between this histone mark and the histone variant, as indicated earlier.

This is in marked contrast to what has been observed for genes that are covered by facultative heterochromatin. Here, the incorporation of FfH2A.Z has a clear effect on the + 1-nucleosome positioning. As depicted in Fig. 8B, a strong positioning of the + 1-nucleosome in genes marked with FfH2A.Z and H3K27me3 is observed (left panel) resulting in a highly accessible NDR, which may cause gene transcription in otherwise silent genomic regions. Indeed, this phenomenon is dependent on the presence of the histone variant FfH2A.Z (Fig. 8B, right panel). These results are supported by the finding that genes covered with H3K27me3 show a weaker positioning of the + 1-nucleosome (Fig S10 left panel) and thus could result in the inaccessibility of the NDR and gene silencing. A similar pattern is observed for all non-FfH2A.Z (< 7.5 CPM in 5’ region) genes, where no distinct + 1-nucleosome positioning is observed (Fig. S10 right panel). Thus, the obtained data point towards a stabilizing effect of FfH2A.Z on the + 1-nucleosome positioning, but further investigations are required to unequivocally connect FfH2A.Z incorporation with the stabilization of the + 1-nucleosome.

### FfH2A.Z is crucial for wild type-like fungal development in *F. fujikuroi*

As already shown, the underrepresentation of FfH2A.Z but not overexpression resulted in a major transcriptional imbalance in *F. fujikuroi*. Next, we asked about the role of FfH2A. Z in fungal development is also in this fungus. While, for the yeast *S. cerevisiae*, lack of ScH2A.Z resulted in a reduction in fungal fitness [75], for the ascomycete fungi *F. graminearum* and *N. crassa* absence of H2A.Z led to adverse developmental defects [29, 30].

**Fig. 9:**
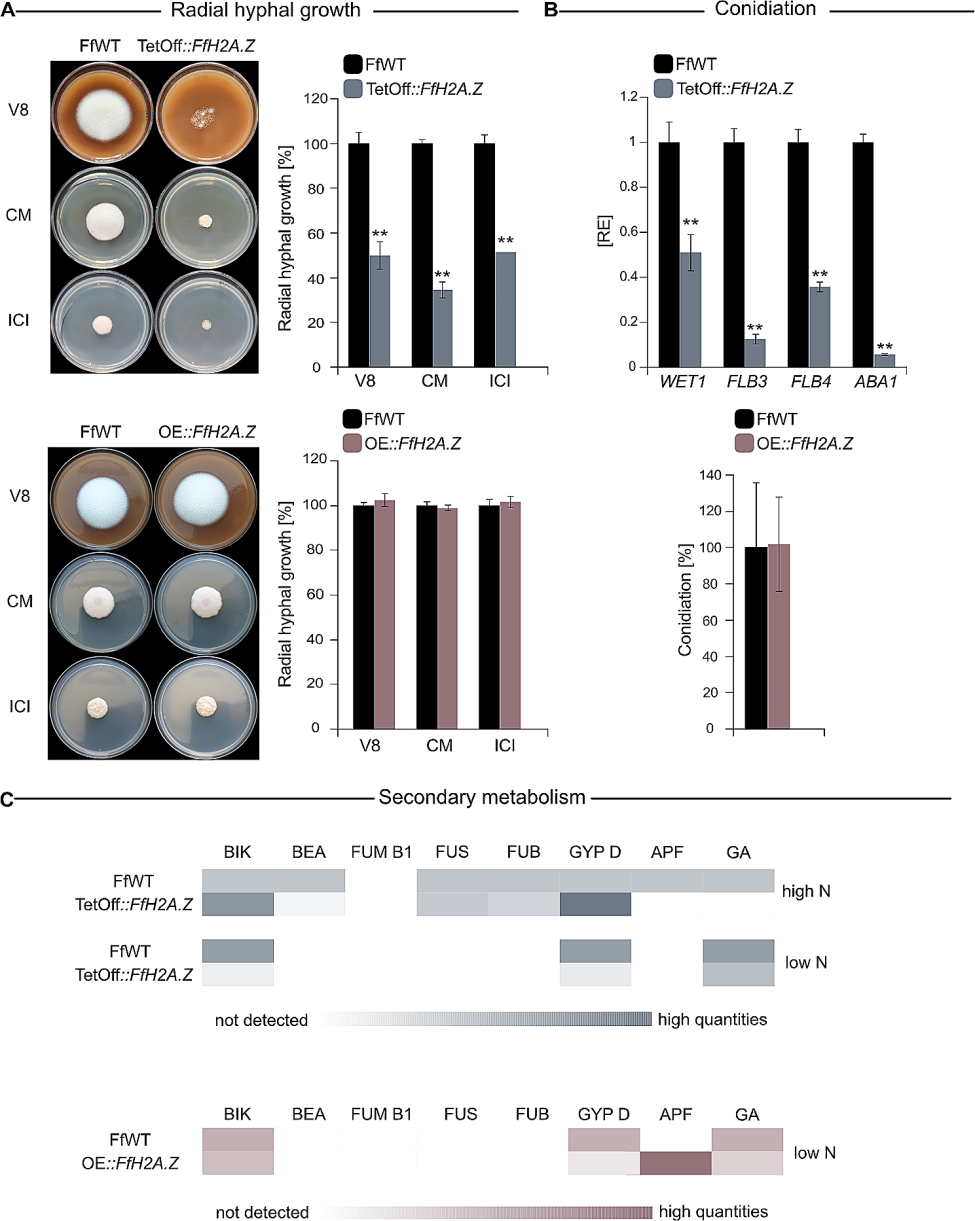
Characterization of *FfH2A.Z* knock-down and overexpression in fungal development. (**A**) Radial hyphal growth assay on complete media (V8, CM) and minimal medium (ICI supplemented with 6 mM glutamine). Strains were grown for 4 days (TetOff::*FfH2A.Z*) and 5 days (OE::*FfH2A.Z*) at 30 °C, respectively. Experiments were performed in biological triplicates. Hyphal growth of *Fusarium fujikuroi* (FfWT) was arbitrarily set to 100%. Mean values and standard deviations are shown. For statistical analysis, a student’s *t*-test was performed. Asterisks above the bars denote significant differences in vegetative growth of the indicated strains compared to FfWT, *p < 0.05; **p < 0.001. (**B**) Expression of the conidiation-specific genes *FfWET1*, *FfFLB3-4*, and *FfABA1* in FfWT and *FfH2A.Z* knock-down mutant were assessed 4 dpi after inoculation on V8 supplemented with doxycycline hyclate (DOX). Plates were incubated in 16 h light and 8 h dark at 20 °C. Expression levels were monitored using RT-qPCR. Mean values and standard deviations are shown and RE denotes relative expression. The conidiation assay was performed using FfWT and OE::*FfH2A.Z*. V8 plates inoculated with the respective strains were incubated for 7 days at an L/D rhythm before harvesting and conidia quantitation. Experiments were performed in triplicates. Conidia production of FfWT was arbitrarily set to 100%. A student’s *t*-test was performed to assess the significance levels. Asterisks above the bars denote significant differences in the gene expression/conidia production of the indicated strains compared to the respective wild type, *p < 0.05; **p < 0.001. (**C**) Heatmap of the relative SM production of *FfH2A.Z* knock-down and overexpression strains compared to FfWT in different nitrogen conditions. *Fusarium* metabolites were analyzed by LC-HRMS after cultivation in liquid ICI supplemented with 60 or 6 mM glutamine (30 °C, 180 rpm). To ensure result comparability, the analysis of all mutants and cultivation conditions was performed in three independent replicates. Determined quantities are normalized to the biomass formation (area/g dry weight). SM biosynthesis of FfWT was arbitrarily set to 100%

To assess the fungal growth behavior in more detail, a plate assay on V8 and CM (complete media) as well as synthetic ICI supplemented with 6 mM glutamine as the sole nitrogen source (minimal medium) was performed. Fungal growth of TetOff::*FfH2A.Z* mutant strains was assessed under the addition of DOX. Plates were inoculated with 1,000 conidia each of FfWT, TetOff::*FfH2A.Z*, or OE::*FfH2A.Z*. Plates were grown 4 and 5 days post inoculation (dpi) for knock-down and overexpression, respectively at 30 °C in the dark. Growth was significantly impeded on all tested media conditions for TetOff::*FfH2A.Z* to approximately 50%, 50%, and 35% for V8, ICI, and CM, respectively (Fig. 9A). Next to this, mycelial growth appeared to be completely aberrant and was barely able to overcome the stage of germination on V8. In contrast, *FfH2A.Z* overexpression did not lead to any differences in radial hyphal growth on all tested media (Fig. 9A). Attenuated growth was also observed for all *FgH2A.Z* deletion strains but not overexpression strains in *F. graminearum* [29]. Similar to *Fusarium*, retarded growth was also observed in *NcH2A.Z* deletion strains in *N. crassa* [25, 30].

Asexual reproduction is important for most fungal pathogens to disperse the first inoculum before plant colonization. Conidiospore production of TetOff::*FfH2A.Z* and OE::*FfH2A.Z* was assessed on solid V8 for 4 and 7 dpi, respectively. As the *FfH2A.Z* knock-down strains displayed an unstable growth phenotype, when inoculated with an agar plug on V8, conidia production could not be quantified. Since inoculation with conidia appeared to be more stable, cultivation of FfWT and TetOff::*FfH2A.Z* conidia on solid V8 supplemented with DOX for 4 days at 20 °C under L/D conditions was performed. Mycelia were then harvested and tested for the expression of the already well-characterized fungal condition-specific genes *WET1*, *FLB3*, *FLB4*, and *ABA1* in *F. fujikuroi* and *Aspergillus nidulans* [70, 76]. Briefly, the transcription factors *FfFLB3*, *FfFLB4*, and *FfABA1* are crucial for fungal conidia formation, while *FfWET1* seems to be involved in conidia maturation in *F. fujikuroi* [70]. All tested genes were significantly downregulated (Fig. 9B), suggesting hampered conidiogenesis and an important role for FfH2A.Z in the wild type-like formation of conidia. These results are in accordance with *N. crassa* and *F. graminearum*, where the loss of H2A.Z was reported to result in impeded conidia production in these species [29, 30]. The conidiospore production in the *FfH2A.Z* overexpression strains was wild type-like (Fig. 9B) as observed for OE::*FgH2A.Z* in *F. graminearum* [29]. Unfortunately, ChIP-seq analyses were performed under conidia non-inducing conditions (*FfABA1* and *FfWET1* are not transcribed, Table S1), thereby prohibiting to draw further conclusions.

Fusaria are well-known for their huge portfolio of SMs [6, 7]. NP biosynthesis is not essential *per se* but can provide a selective advantage for the fungus in some environmental niches [8, 77]. Manipulation of the chromatin landscape by the deletion or overexpression of genes encoding certain histone-modifying enzymes, ATP-dependent chromatin remodelers, or other factors has already been demonstrated to alter SM gene expression [16]. Hence, to investigate if FfH2A.Z is also involved in SM gene regulation, the production of known fusarial SMs was monitored in liquid ICI supplemented with 60 or 6 mM glutamine for TetOff::*FfH2A.Z* and 6 mM glutamine for OE::*FfH2A.Z*. Fungal cultures were incubated for 7 dpi at 30 °C in the dark and analyzed *via* LC-HRMS. The stability of the TetOff::*FfH2A.Z* strains was confirmed by western blot (Fig. S11). Knock-down of *FfH2A.Z* predominantly resulted in decreased biosynthesis of selected SMs under different culture conditions (Fig. 9C). In detail, cultivation in high nitrogen revealed slightly increased bikaverin (BIK) and gibepyrone D (GYP D) biosynthesis, while beauvericin (BEA) and apicidin F (APF) were nearly abolished. APF and BEA biosynthesis are normally strongly induced under the addition of sufficient nitrogen [78, 79], while BIK biosynthesis is restricted to low nitrogen supplementation and acidic pH [80, 81]. Next to this, basal levels of gibberellins (GA) were determined in the FfWT strain under normally GA-repressing conditions [82, 83], which are not present in the TetOff::*FfH2A.Z* supernatant. Supplementation of only low amounts of nitrogen led to drastically decreased biosynthesis of BIK and GYP D in liquid culture, which is consistent with the transcriptional data. For BIK, FfH2A.Z is absent from the key enzyme *FfBIK1* (FFUJ_06742) but incorporated along the gene bodies of two further cluster genes, *i*.*e*., *FfBIK5* (FFUJ_06745) and *FfBIK7* (FFUJ_06747) a transcription factor enhancer and multidrug facilitator superfamily (MFS) transporter, respectively (Fig. S12A). For GPY D, two small peaks indicating the presence of FfH2A.Z, are observed near the TSS of *FfGPY1* (FFUJ_12020) and *FfGPY2* (FFUJ_12021), the SM key enzyme, and the cluster-specific transporter, respectively (Fig. S12B). It is tempting to speculate that the removal of FfH2A.Z at these loci, led to the downregulation of the associated genes and in consequence, to the drastically decreased biosynthesis of these compounds. SM biosynthesis was also imbalanced in the OE::*FfH2A.Z* strains. Here, increased production of APF was observed, while GYP D and GA biosynthesis was slightly reduced. In detail, APF biosynthesis is normally restricted to high nitrogen [78]. Unfortunately, the transcriptomic data does not confirm elevated expression of the APF cluster genes (FFUJ_00003-FFUJ_00013) in the OE::*FfH2A.Z* strains. This discrepancy could be explained by the fact that for transcriptomic analysis, mycelium was harvested at 3 dpi, while for SM analysis, samples were taken at 7 dpi. For APF biosynthesis in *APF*-repressing conditions (6 mM glutamine), not surprisingly, no relevant FfH2A.Z peak was observed. Similar to the observations in *F. fujikuroi*, the *FgH2A.Z* deletion mutants were able to produce deoxynivalenol in inducing conditions only in basal amounts, while biosynthesis of the OE::*FgH2A.Z* strain was wild type-like [29].

The *FfH2A.Z* overexpression strains were additionally tested for their ability to colonize rice plants since a slight decrease in GA biosynthesis in axenic culture was observed. Freshly germinated rice seedlings were infected with the OE::*FfH2A.Z* strain, which exhibited wild type-like virulence as depicted in Fig. S13. Unfortunately, the unstable growth of the TetOff::*FfH2A.Z* strain prohibited attempts to perform virulence assays in this background.

To sum up, FfH2A.Z appears to be essential for fungal development as well as to facilitate wild type-like fungal secondary metabolism in *F. fujikuroi*. The direct role of FfH2A.Z in fungal SM biosynthesis is questionable, despite the presence of FfH2A.Z at some regulatory genes at deregulated BGCs. A closer look at the FfH2A.Z occupancy in non-deregulated BGCs in normally inducing conditions revealed that the GA as well as fumonisin (FUM) BGCs are devoid of the variant. The presence of FfH2A.Z in these conditions, at the silent fusaric acid BGC shows FfH2A.Z occupancy also in regulatory elements such as transcription factors, and key enzymes (Fig. S14) albeit with no gene expression. It is noteworthy to mention that even though there is more FfH2A.Z present in the OE::*FfH2A.Z* mutant strain on the protein level, we cannot provide evidence that FfH2A.Z is indeed incorporated to a higher extent in the nucleosome structure, which could explain the subtle phenotype observed in this strain.

## Conclusion

Chromatin is the basic packaging form of eukaryotic DNA and as such serves as a substrate for the transcriptional machinery. To ensure the timely coordinated expression of the underlying genes, multifaceted mechanisms are at play in safeguarding this process. Histone variants are non-canonical histones, which can be exchanged against regular canonical histones and modify the chromatin landscape and in consequence, the transcriptional output. The prominent histone variant H2A.Z is well conserved from yeast to humans and is described to govern fundamental roles in gene regulatory processes such as gene silencing but also gene transcription. As its molecular functions are described to be manifold, the overall incorporation of the histone variant H2A.Z is not well understood. In this study, we shed light on the yet unexplored regulatory roles of the histone variant FfH2A.Z in the phytopathogen *F. fujikuroi*, the founding member of the FFSC. As described in other eukaryotes, incorporation of FfH2A.Z is largely restricted to the + 1-nucleosome in *F. fujikuroi*. GO-enrichment analysis revealed that FfH2A.Z is enriched at loci which are associated with gene regulatory processes, which may promote gene transcription. In line with this, depletion and overexpression mutants of *FfH2A.Z* are impaired in fungal development and secondary metabolism, emphasizing its fundamental function in gene regulatory processes also in this species. Next, we showed the global distribution of two already well-characterized histone marks, *i*.*e*., trimethylation of H3K4 and H3K27, associated with gene transcription and silencing, respectively, and their correlation with FfH2A.Z. Unlike as observed in other model systems, our data indicate no clear relationship between the presence of FfH2A.Z and H3K4me3 in regard to the transcriptional output, but the incorporation of FfH2A.Z results in gene transcription at otherwise silent facultative heterochromatic loci (H3K27me3), which may be caused by the strong positioning of the + 1-nucleosome. To the best of our knowledge, we show here for the first time, that incorporation of the non-canonical histone FfH2A.Z is crucial for gene transcription regardless of the chromatin state. Overall, our data point towards an independent role of FfH2A.Z and thus no crosstalk between FfH2A.Z and the prominent histone marks, H3K4me3 and H3K27me3, in this phytopathogen.

## Materials and methods

### Fungal strains and culture conditions

The *F. fujikuroi* IMI58289 wild-type strain (FfWT) provided by Commonwealth Mycological Institute, Kew, UK, was used as a parental strain to generate the *FfH2A.Z* knock-down strain (TetOff::*FfH2A.Z*), the constitutive *FfH2A.Z* overexpression (OE::*FfH2A.Z*) strain as well as the N- and C-terminal hemagglutinin (HA)-tagged *FfH2A.Z* (*HA*::FfH2A.Z, FfH2A.Z::*HA*) strains. Fungal growth on solid media was induced on V8 media (30 mM CaCO_3_, 20%, v/v, vegetable juice; Campbell Food, Puurs, Belgium), Complete Medium [84], and synthetic ICI minimal media (Imperial Chemical Industries Ltd., London, UK, [85] supplemented with 6 mM glutamine (Carl Roth). Plates were inoculated either with a 5 mm agar plug or 1,000 conidia each and incubated at 30 °C in constant darkness for 5 days. In the case of the TetOff::*FfH2A.Z* mutant strains, media was supplemented with either 0-100 µg/mL doxycycline hyclate (DOX, Sigma-Aldrich) to induce silencing of *FfH2A.Z*. For gDNA extraction and RNA isolation, fungal mycelia were harvested from solid CM media covered with a cellophane sheet (Folia Bringmann) after incubation for 2–3 days at 30 °C in the dark. V8 media were inoculated with a 5 mm agar plug and incubated in the presence of 18 h light and 6 h darkness (L/D), 20 °C, and 70% humidity to trigger conidiospore production. Conidia production was assessed using a Neubauer-improved cell counting chamber under a light microscope (Carl Zeiss). Submerged fungal growth was induced in 100 mL Darken pre-culture medium [86] on a rotary shaker at 180 rpm, 30 °C in constant darkness for 3 days. To trigger SM production in the OE::*FfH2A.Z* and N- and C-terminal FfH2A.Z tagged strains, 0.5 mL of pre-culture was transferred to 100 mL liquid ICI media supplemented with 60 mM (high nitrogen) or 6 mM (low nitrogen) glutamine as sole nitrogen source. Cultures inoculated with the TetOff::*FfH2A.Z* knock-down strain were induced under the supplementation of 0–50 µg/mL DOX and high or low nitrogen. Cultivation was performed as described earlier [87]. Flasks were incubated on a rotary shaker (180 rpm, 30 °C) in the dark. Mycelia were harvested 3–4 days post inoculation (dpi) for RNA isolation and protein extraction, or 7 dpi for SM analysis.

### Plasmid construction

To assemble the *FfH2A.Z* silencing (TetOff::*FfH2A.Z*) and constitute *FfH2A.Z* overexpression (OE::*FfH2A.Z*) plasmids, yeast recombination cloning (YRC) was performed as described earlier [88, 89]. All primers (Merck Millipore) are listed in Table S4. DNA fragments used for cloning were amplified using a high-fidelity DNA polymerase (Q5-polymerase, NEB® or Phusion High-Fidelity DNA polymerase, Thermo Scientific). For the TetOff cloning, ca. 1 kb flanks upstream and downstream of the *FfH2A.Z* start codon were amplified with primer pairs H2A.Z_TET_5F/H2A.Z_TET_5R and H2A.Z_TET_3F/H2A.Z_TET_3R, respectively. The TetOff construct (PgpdA-tTA-TcgrA-TetO7-Pmin) was amplified from pFW9 [49] with primers hph_PgpdA_F/TET_off_R. For selection, the hygromycin resistance gene (hygR) including the TtrpC terminator was amplified from pCSN44 [90] with hph_F/TtrpC_R. The *Xba*I/*Hin*dIII-digested shuttle vector pYES2 (Life Technologies) was used as the backbone. For the construction of the *in loco* overexpression of *FfH2A.Z*, approximately 1 kb upstream (5’) as well as the native *FfH2A.Z* gene sequence followed by 1 kb downstream (3’) were amplified from FfWT genomic DNA (gDNA). The *Sac*II/*Eco*RI-digested shuttle vector pNAH-OGG served as a template for the amplification of the strong constitutive olic promotor (Polic) from *Aspergillus nidulans* followed by the hygR [88]. The *Eco*RI/*Xho*I-digested shuttle vector pRS426 was used as the backbone for the overexpression plasmid [91]. The competent yeast strain *S. cerevisiae* FY834 was used to assemble the construct [92].

The plasmids harboring the N- and C-terminal HA-tagged *FfH2A.Z*, respectively, were cloned using the NEBuilder® HiFi DNA Assembly Kit (NEB®). Briefly, the plasmid backbone was amplified from the high-yield shuttle vector pRS426 using the primer pair Primer-3R/ HiFi-AmpR-p5-R. The 3xHA sequence was synthesized by Merck Millipore and directly used for the assembly. Both fragments served as an insert for the assembly of the N- and C-terminal tagged FfH2A.Z plasmid. For the C-terminal tagged version, the 5’ region including the native *FfH2A.Z* gene (FFUJ_01849) and the downstream region was amplified from FfWT gDNA with the primer HiFi-H2AZ-HU-5 F and HiFi_C-HA-H2A.Z_R and HiFi-H2AZ-HD-pTrpc/HiFi-H2AZ-HD-3R, respectively. The artificial glucanase terminator sequence from *Botrytis cinerea* (BcTgluc) coupled to the nourseothricin resistance cassette (natR) was amplified from p*SET1*^*C*^ [70] using the primers C-HA-Tgluc_F/ HPH-F. Similarly, for the N-terminal tagged HA::*FfH2A.Z* plasmid, the 5’ fragment, as well as the native *FfH2A.Z* gene, were obtained from FfWT gDNA using the primer pairs HiFi-H2AZ-HU-5 F/HiFi-H2A.Z-BH_R and HiFi-H2A.Z-HHA_F/ H2A.Z-BcTgluc_R, respectively. The BcTgluc terminator and natR resistance cassette were again amplified from p*SET1*^*C*^ with the primers Tgluc-F2 and HPH-F. The plasmids were assembled according to the manufacturer’s procedure. To propagate plasmid DNA (pDNA) the high-efficiency *Escherichia coli* strain DH5α (NEB®, NEB 5-alpha) was transformed as described in the manufacturer’s protocol. The assembled plasmids were sequenced (LGC Genomics, Germany) to ensure the correctness of the inserts. All primers used for sequencing are listed in the S4 Table.

### Fungal transformation

*F. fujikuroi* protoplast generation and fungal transformation were performed as described elsewhere [93]. Briefly, PCR-amplified TetOff::*FfH2A.Z* (H2A.Z_TET_5F/H2A.Z_TET_3R) (Fig. S5A), or 10 µg of enzymatically digested pDNA, *i*.*e*., OE::*FfH2A.Z* (*Ehe*I/*Bgl*I) (Fig. S6A), HA::*FfH2A.Z* and *FfH2A.Z*::HA (*Bsa*I/*Sca*I) (Figs. S2A and S3A), was used for the transformation of FfWT protoplasts. The obtained transformants were selected on regeneration media supplemented with either 100 ppm hygromycin B (Merck Millipore) or 100 ppm nourseothricin (Jena Bioscience), depending on the appropriate resistance marker. In situ homologous recombination events for TetOff::*FfH2A.Z* (Fig. S5B), OE::*FfH2A.Z* (Fig. S6B), HA::*FfH2A.Z* (Fig. S2B) and *FfH2A.Z*::HA (Fig. S3B) were verified by diagnostic PCR using the GoTaq® G2 DNA Polymerase (Promega). Briefly, to verify the correct integration of the TetOff::*FfH2A.Z* construct H2A.Z_TET_5dia/TtrpC_dia and H2A.Z_TET_3dia/TET_dia were used. The absence of the native wild-type gene was shown using the primers H2A.Z_TET_5dia/H2A.Z_TET_WTdia. In situ integration of the *FfH2A.Z* overexpression construct was verified by the use of the primer pairs H2A.Z_diaF/trpC_T, trpc_P/H2A.Z_Wtdia_R as well as H2A.Z_Wtdia_F/H2A.Z_diaR. For both, N- (HA::*FfH2A.Z*) and C-terminal (*FfH2A.Z*::HA) tagged FfH2A.Z strains, in situ complementation was shown with the primers H2A.Z_diaTagF paired with Bcgluc_seqR and Tgluc_hiF combined with H2A.Z_diaTagR. Again, the absence of the native wild-type gene was shown using the primers H2A.Z_diaTagF/H2A.ZdiaTagR. To ensure the functionality of all transformed constructs the *FfH2A.Z* gene expression (FFUJ_01849) was assessed in all transformants (Figs. 3 and S5C and S6C).

### Standard molecular techniques

Fungal gDNA extraction from lyophilized and ground mycelia was performed as described elsewhere [94]. Competent *S. cerevisiae* and *E. coli* strains were cultured as described earlier [88]. Yeast pDNA was extracted with the GeneJET Plasmid Miniprep Kit according to the manufacturer’s protocol. Next to this, pDNA from the *FfH2A.Z* silencing and overexpression strains were propagated using the *E. coli* DH5α strain (Invitrogen™) and pDNA was extracted using the GeneJET Plasmid Midiprep Kit (Thermo Fisher Scientific).

### Western blot analysis

Total protein extraction from lyophilized fungal mycelia was performed using 12% (w/v) trichloroacetic acid (TCA) as described previously [95]. For western blot analysis, approximately 15–50 µg of total protein extracts were separated on a SDS gel and blotted onto a positively charged nitrocellulose membrane (Amersham™ Protran®). The membranes were probed with either an anti-H3 C-Term- (Active motif, 91299), an anti-H2A.Z- (Active motif, 39648), an anti-H3K27me3- (Active motif, 39155) or an anti-H3K4me3- (CellSignal, 9751) specific antibody. For the chemiluminescence signal detection, all already probed membranes were incubated with an anti-rabbit HRP-conjugated secondary antibody. Then, western blot membranes were developed using the Clarity™ ECL Western Substrate (Bio-Rad) and signals were quantified with a ChemiDoc™ XRS system (Bio-Rad). The ImageJ software was used to perform densitometric analysis. All signals were normalized to the histone H3 C-term signal. For reference, the FfWT signal ratio was set to 1.

### Expression analysis by quantitative RT-qPCR and high throughput RNA-sequencing analysis

Isolation of fungal RNA was performed with lyophilized and ground mycelia from cultivation on solid CM or under liquid standard conditions (ICI supplemented with 6 or 60 mM glutamine) for 3–4 days. Total RNA was extracted from samples, using the RNA reagent TRIzol (Thermo Fisher Scientific) as described in the manufacturer’s manual. To facilitate cDNA synthesis, 2 µg RNA was treated with DNaseI (Thermo Fisher Scientific) and then reversely transcribed into cDNA using the LunaScript™ RT SuperMix Kit (NEB®). The iTag™ Universal SYBR® Green Supermix (Bio-Rad) and the iCycler iQ Real-Time PCR System (Bio-Rad) were used for expressional profiling. Primer efficiency was set between 90 and 110%. The ∆∆Ct method [96] was applied to evaluate the obtained results. The obtained data were normalized with the housekeeping genes actin (FFUJ_02611), glyceraldehyde-3-phosphate dehydrogenase (GPD, FFUJ_13490), and β-tubulin (FFUJ_07385). Primers used for RT-qPCR are listed in Table S4.

For RNA-sequencing, mycelia were kept for exactly 3 days in liquid ICI supplemented with 6 mM glutamine as sole nitrogen source. RNA extraction was performed as described above, utilizing the TRIzol reagent. Quality control, library preparation, and sequencing of the samples were performed by the Vienna BioCenter Core facilities (Vienna, Austria). All experiments were performed in duplicates. Library prep and sequencing were accomplished using a poly-A enrichment kit (NEB) and Nextera Library prep kit. 50 bp single-end sequencing was performed using a HiSeq v4 Illumina sequencer. Obtained sequences were de-multiplexed, quality controled, filtered using trimmomatic 0.36 [97], and mapped on the already available FfWT genome assembly [12]. Mapping was performed using BWA [98] and reverse transcripts were counted using Python script HTSeq [99]. Normalization and statistics were done using R/Bioconductor and the limma and edgeR packages, using mean-variance weighting (voom) and TMM normalization [100]. A significance cut-off of *p* < 0.01 and differential expression of +/-1 (2-fold) was applied for analysis. Transcription levels are log2 read counts per kilobase of exon per million library reads (RPKM). The RNA-seq data has been deposited in NCBI’s Gene Expression Omnibus [101] and is accessible through the GEO Series accession number GSE237765. GO annotation was performed using PANNZER2 [102] and processed using the R/Bioconductor library “Gostats”, “GSEABase”; GO term reduction was done using library “rrvgo”. Only GO terms enriched in the selected gene sets with *p* < 0.05 were considered for further investigation. The FfH2A.Z occupation was defined by the maximum normalized coverage in the regions 200 bp upstream and 600 bp downstream of ATG of genes being greater than 15 in both replicates. Significant up- or down-regulation between TetOff::*FfH2A.Z* and FfWT was considered by a log-difference of |1| and a *p*-value < 0.01.

#### BLAST search

For finding homologs of the *F. fujikuroi* IMI58289 proteome we used NCBI-BLAST with the following settings: max e-value 1E^− 10^ and max sequence hits 500 (default). The results were later filtered to a percentage identity above 40%. The search was run against the database refseq_protein version “2024-01-13T00:00:00”. Based on the visual interpretation of histograms from the different categories (e.g. FfH2A.Z decorated, H3K27me3 decorated) we considered protein sequences with less than 50 hits low conserved, sequences with more tha 300 hits were considered highly conserved.

### Chromatin immunoprecipitation and sequencing

Chromatin immunoprecipitation and subsequent sequencing (ChIP-seq) was performed as described previously [69, 103]. Fungal mycelia were cultivated for three days in liquid ICI (6 mM glutamine) before crosslinking with 1% (v/v) formaldehyde. For immunoprecipitation, sonicated mycelia were incubated either with an anti-HA (Abcam, 9110), an anti-H3K27me3- (Active motif, 39155), an anti-H3K4me3- (CellSignal, 9751) specific antibody. The assay was performed in biological duplicates, including input controls for every strain. The quality control, library preparation, and sequencing of the samples were performed by the Vienna BioCenter Core Facilities (Vienna, Austria). Paired-end sequencing was performed using a HiSeq v4 Illumina sequencer and quality filtering, trimming, and mapping were performed similarly as described for RNA-seq experiments. Quantification of mapping in specific regions around genes was performed in R using the GenomicRanges Biostrings [104, 105]. Regions of interest and ChIP coverage were determined as follows: For H3K27me3, the maximum coverage in the region 100 bp upstream and 1,000 bp downstream of ATG of each gene, coverages above 15 were considered as positives. For FfH2A.Z in the region 600 bp upstream and 200 bp downstream of ATG of each gene with threshold 15 CPM, and H3K4me3 max coverage upstream 200 bp, downstream 600 bp from ATG > 20 CPM. All thresholds were determined by visually checking the peak background relationships in each experiment. Peak-calling of FfH2A.Z was done in R, functions used are available *via* Github (https://github.com/symbiocyte/MNase). The ChIP-seq data have been deposited in NCBI’s Gene Expression Omnibus [101] and is accessible under GSE237765.

### Nucleosome mapping

The nucleosome landscape in FfWT was recorded essentially as described recently [106]. Briefly, readily crosslinked and quick-frozen mycelia (3 days, liquid synthetic ICI, 6 mM glutamine) was treated with Micrococcal Nuclease I (MNase I) (60 U/µL, Thermo Fisher Scientific) for 4 min. The reaction was stopped by the addition of 0.5 M EDTA (final conc. 40 mM EDTA). Reverse cross-linking of digested DNA was performed for at least 30 min at 65 °C. Subsequently, samples were diluted and treated with proteinase K (1 h and 40 °C, Thermo Fisher Scientific) for protein degradation. DNA fragments were extracted using phenol and separated on an agarose gel to check for successful MNase I digestion. Fragmented DNA was cut from the gel at 150 bp and extracted using the Monarch® DNA Gel Extraction Kit (NEB®). Quality control, library preparation, and sequencing were performed at the Vienna BioCenter Core Facilities (Vienna, Austria). Mapping files were filtered for fragment sizes 130–170 bp, coverage per bp was calculated using R/GenomicRanges [104], and + 1- or -1-nucleosomes were identified using functions find_plusOne_nucleosome or find_minusOne_nucleosome (see Github page above). The peak height was located as follows: The positions of peaks were identified by using the chromosomal coverage traces and applying a differential filter of size 151: (1/150)_75_0(-1/150)_75_ followed by the detection of the position when the resulting trace descents through zero, indicating positive peaks. At the resulting chromosomal positions, the height from the original coverage file was collected. The + 1-nucleosome was defined as the nucleosome flanking the NDR in the direction of the gene ATG and was determined as follows: To find the + 1-nucleosome the outcome of locate_peak_hight (positions and heightheight of nucleosomal peaks), the coverage traces per nucleosome, and the genomic positions of genes were used. A cutoff of a minimal peak height of 20 reads per nucleotide was applied. Starting 100 bp downstream of the ATG of annotated genes, we searched the NDR until 2 kb upstream of the ATG. The distances between peaks have to be larger than 200 bp, the NDR proximate nucleosome has higher coverage than 20 reads, and the number of nucleotides in the NDR with read coverages lower than the minimum coverage + 0.1*max.cov-min.cov of the region between nucleosome peaks must be larger than 20 bp. Next to this, the − 1-nucleosomes were detected similarly by identifying the NDR flanking nucleosome facing away from the gene start. The R functions are available at Github (https://github.com/symbiocyte/MNase).

### SM production and chemical analysis

Fungal supernatant was retrieved from 7-day-old cultures of liquid synthetic ICI supplemented with high nitrogen (60 mM glutamine) or low nitrogen (6 mM glutamine) incubated on an orbital shaker at 180 rpm and 30 °C. For the analysis using liquid chromatography-high-resolution mass spectrometry (LC-HRMS), 500 µL of the cultures were combined with 500 µL of acetonitrile (MeCN) and subjected to extraction in an ultrasonic bath for 20 min. Following filtration through a syringe filter (RC, 0.2 μm pore size, Phenomenex), the LC-HRMS analysis was performed using a Nexera XR LC system (Shimadzu, Duisburg, Germany) linked to an SPD-M30A Diode Array Detector (Shimadzu, Duisburg, Germany) and an LTQ Orbitrap XL™ mass spectrometer, employing heated-electrospray ionization (HESI) (Thermo Fisher Scientific, Dreieich, Germany) in both the positive and negative ionization modes. Chromatographic separation was performed with a binary gradient composed of MeCN (solvent **A**) and H_2_O (solvent **B**), both containing 0.1% (v/v) FA on a ReproSil Gold C18 column (150 × 2 mm, 3 μm, Dr. Maisch, Ammerbuch, Deutschland). The gradient started with 10% MeCN + 0.1% FA and 90% H_2_O + 0.1% FA for a duration of 2 min and a flow rate of 0.3 mL/min and 40 °C column oven temperature. Over 20 min, solvent **A** was linearly increased to 100%. Subsequently, solvent **A** was maintained at 100% for the ensuing 5 min. Equilibration was conducted for 5 min under the initial conditions. The source voltage was set to 3 kV, while the capillary and vaporizer temperatures were set to 375 °C and 350 °C. The sheath gas flow was set to 40 arbitrary units, auxiliary gas flow to 20 arbitrary units, and sweep gas flow to 10 arbitrary units. Full scans were recorded in centroid mode with a resolution of 30,000 in a mass range of *m*/*z* 80–800. For data acquisition, the Tune Plus 2.7 and Xcalibur 3.1 software (Thermo Fisher Scientific, Dreieich, Germany) were used. Data analysis was performed using the open-source application Skyline 22.2 [107] (MacCoss Lab, University of Washington). A transition list consisting of the exact masses, molecular formulas, and retention times (verified through the injection of reference standards) was employed as identification criteria, and the integration of peak areas was manually validated.

### Rice virulence assay

To test for fungal virulence, the rice cultivar *Oryza sativa* spp. *japonica* cv. Nipponbare, which was kindly provided by the USDA ARS Dale Bumpers National Rice Research Center, Arkansas USA, was used. Infection of already surface-sterilized and germinated seedlings of FfWT, OE::*FfH2A.Z* as well as FfH2A.Z::*HA* was performed as described prior [87]. Rice germlings supplied with 50 µg GA_3_ or water served as a positive or negative control, respectively.

### Electronic supplementary material

Below is the link to the electronic supplementary material.

**Additional file 1: Table S1.** Transcriptome of TetOff::*FfH2A.Z* and OE::*FfH2A.Z* in comparison to the *F. fujikuroi* wild-type strain (FfWT).

**Additional file 2: Table S2.** Gene Ontology (GO) enrichment analysis of down-regulated genes enriched with FfH2A.Z of biological processes

**Additional file 3: Table S3.** Gene Ontology (GO) enrichment analysis of up-regulated genes enriched with FfH2A.Z of biological processes

**Additional file 4: Table S4.** Table of conserved gene analysis including FfH2A.Z marked genes and genes decorated with the histone marks H3K27me3 and/or H3K4me3

**Additional file 5:** Supplementary material

## Data Availability

Sequencing data that support the findings of this study are available from NCBI with the identifier GEO Series accession number GSE237765.
