# Supplementary material for: Incorporation of the histone variant H2A.Z counteracts gene silencing mediated by H3K27 trimethylation in Fusarium fujikuroi
Source: Epigenetics Chromatin. 2024 Mar 20;17:7. doi: 10.1186/s13072-024-00532-y (PMC10953111; doi:10.1186/s13072-024-00532-y)
Supplement: Supplementary file 5 — Additional file 5: Supplementary material [file 13072_2024_532_MOESM5_ESM.pdf]

SUPPORTING INFORMATION

Incorporation of the histone variant H2A.Z counteracts gene silencing mediated by H3K27 trimethylation in *Fusarium fujikuroi*

Anna K. Atanasoff-Kardjalieff, Harald Berger, Katharina Steinert, Slavica Janevska, Nadia Ponts, Hans-Ulrich Humpf, Svetlana Kalinina, Lena Studt-Reinhold

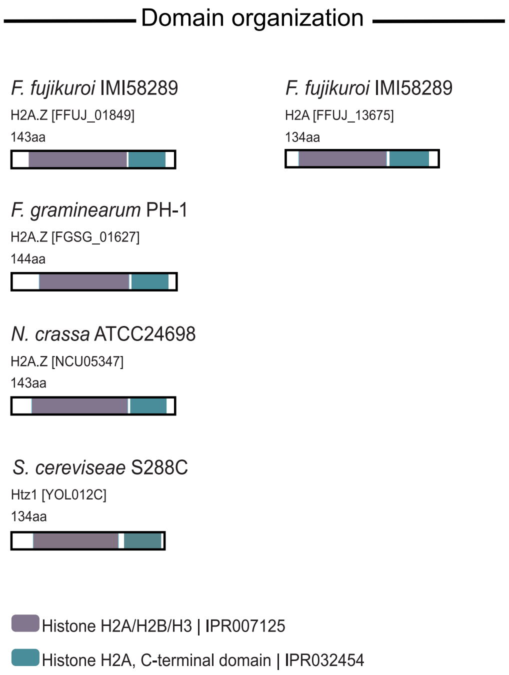

Fig S1 Domain structure analysis of the histone variant H2A.Z and H2A. Graphical representation of the domain structure of *Fusarium graminearum* PH-1 FgH2A.Z and orthologs in other fungal species including *Fusarium fujikuroi*. The conserved histone fold and C-terminal patch are indicated in the figure. The domain structure was determined using the InterPro (1) database. The InterPro accession numbers are shown.

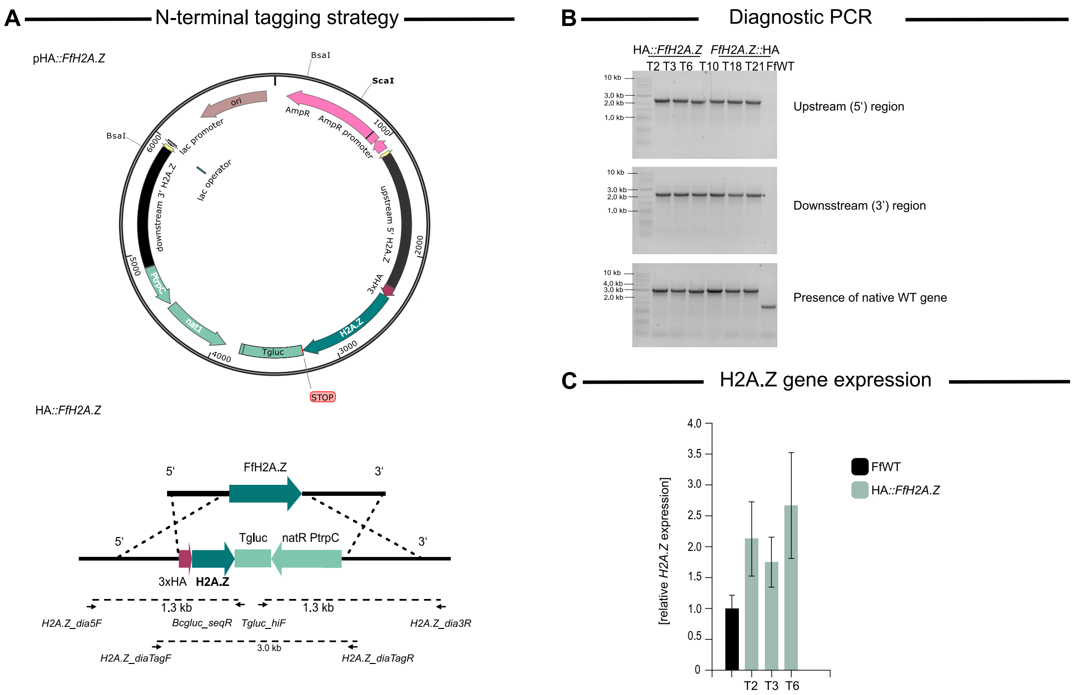

Fig S2 Cloning strategy and verification of the HA::*FfH2A.Z* tagging construct in *Fusarium fujikuroi* (FfWT). **A** Plasmid map of the N-terminal FfH2A.Z (HA::*FfH2A.Z*) tagging construct. *Sca*I and *Bsa*I were used for plasmid linearization prior transformation. Restriction sites are depicted in the plasmid map. Tagging of the native *FfH2A.Z* wild-type gene was performed by homologous recombination. Here, a 3x-hemagglutanin (HA) tag was attached at the N-terminal end of the native *FfH2A.Z* gene. Primers used for verification of successful homologous integration are indicated in the scheme below the plasmid map. **B** Successful integration of the construct was verified *via* diagnostic PCR for the three independent mutants HA::*FfH2A.Z*_T2, T3 and T6 by the presence of the the upstream region (5') using the primer pair H2A.Z_dia5F/BcGluc_seqR and the downstream region (3') using the primers H2A.Z_dia3R and Tgluc_hiF. Presence of the tagging cassette was tested using the primer pair H2A.Z_diaTagF/H2A.Z_diaTagR. As negative control gDNA of FfWT was used. In the case of the presence of the native wild-type gene FfWT gDNA served as control. As size marker the 1 kb Plus DNA ladder (NEB) was used. **C** Tagged strains were tested by RT-qPCR to verify wild type-like *FfH2A.Z* gene gene expession. Gene expression of FfWT was arbitrarily set to 1 and used as reference.

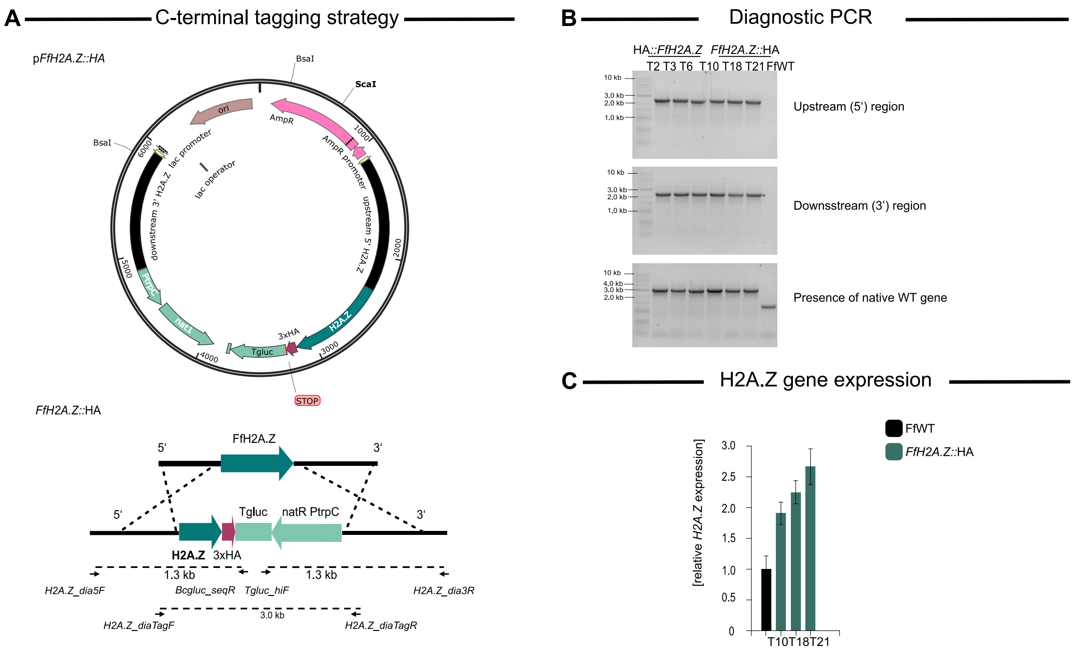

Fig S3 Tagging strategy and verification of the C-terminal FfH2A.Z-tagging construct in *Fusarium fujikuroi* (FfWT). A Plasmid map of the *FfH2A.Z*::HA tagging construct. Before transformation, the plasmid was linearized using the restriction enzymes *Sca*I and *Bsa*I. The restriction sites are depicted in the plasmid map. The histone variant *FfH2A.Z* was taggged C-terminal *via* homologous integration. Therefore, a 3x-hemagglutanin (HA) tag was attached at the C-terminal end of the native *FfH2A.Z* gene. The tagging strategy and primers used for verification of successful homologous recombination are shown in the scheme below the plasmid map. **B** Integration of the tagging construct was verified by diagnostic PCR for all three independent mutants *FfH2A.Z*::HA_T10, T18 and T21. Presence of the the upstream region (5') was tested using the primer pair H2A.Z_dia5F/BcGluc_seqR, while the downstream region (3') was probed using the primers H2A.Z_dia3R and Tgluc_hiF. Presence of the tagging cassette was performed using the primer pair H2A.Z_diaTagF/H2A.Z_diaTagR. As negative control gDNA of FfWT was used. In the case of the native wild-type gene FfWT gDNA served as control. As size marker the 1 kb Plus DNA ladder (NEB) was used. **C** The mutant strains were tested by RT-qPCR to verify wild type-like *FfH2A.Z* gene gene expession. Expressional analysis confirm, that the *FfH2A.Z* is near the wild-type level and not impacted upon tagging. Gene expression of FfWT was arbitrarily set to 1 and used as reference.

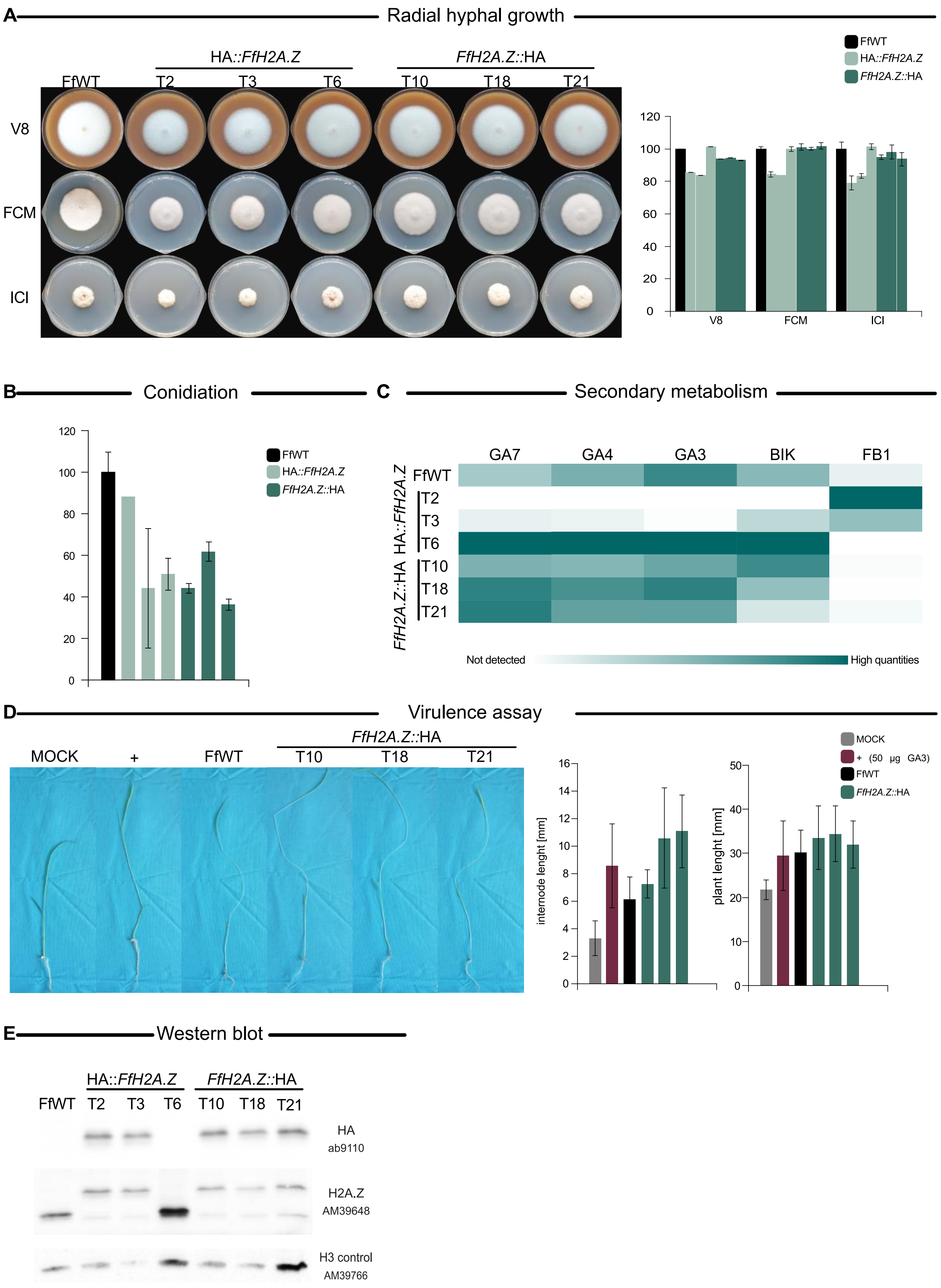

Fig S4 Phenotypization and verification of the N- and C-terminal hemagglutanin (HA)- tagged FfH2A.Z strains in *Fusarium fujikuroi* (FfWT). A Radial hyphal growth assay of N- and C-terminal tagged HA-strains and FfWT on different growth media. As complete media CM and V8 were used, while ICI supplemented with 6 mM glutamine as sole nitrogen source served as minimal media. The different media were inoculated with an agar plug and incubated fo 5 days post inoculation at 30 °C in the dark. Experiments were performed in biological triplicates. Growth of FfWT on the respective media was arbitrarily set to 1. Mean values and standard deviations are shown in

the diagram. **B** Conidiation assay using FfWT, HA::*FfH2A.Z* and *FfH2A.Z*::HA to assess the conidiation behavior. Conidiation was triggered on V8 and samples were incubated for 7 days under a light / dark cycle (18h/6h) at 20 °C and 70 % humidity. Experiments were performed in triplicates. Conidia production of FfWT was arbitrarily set to 1. Mean values and standard deviations are shown in the diagram. **C** Secondary metabolite measurment of routine fusarial metabolites, *i.e.*, the plant hormone giberellic acid (GA), the pigment bikaverin (BIK) as well as the mycotoxin fumonisin (FUM). Strains were grown for 7 days at 30 °C in the dark in synthetic ICI supllmented with 6 mM glutamine as sole nitrogen source. Fungal supernatants were quantified with a LC-HRMS. Quantities of known SMs are illustrated as heatmaps. Determined quantities are normalized to the biomass formation (area/g dry weight). Experiments were performed in biological triplicates and technical duplicates. **D** Virulence assay of infected rice seedlings. The disease progress was assessed 10 days post inoculation. **E** Western blot analysis of FfWT and FfH2A.Z tagging mutant strains. Proteins were probed with an anti-HA specific-, an anti-H2A.Z-specific and an anti H3-C-terminus specific-antibody. For quantification, a densitometric analysis was performed and the respective wild-type strain was arbitrarily set to 1.

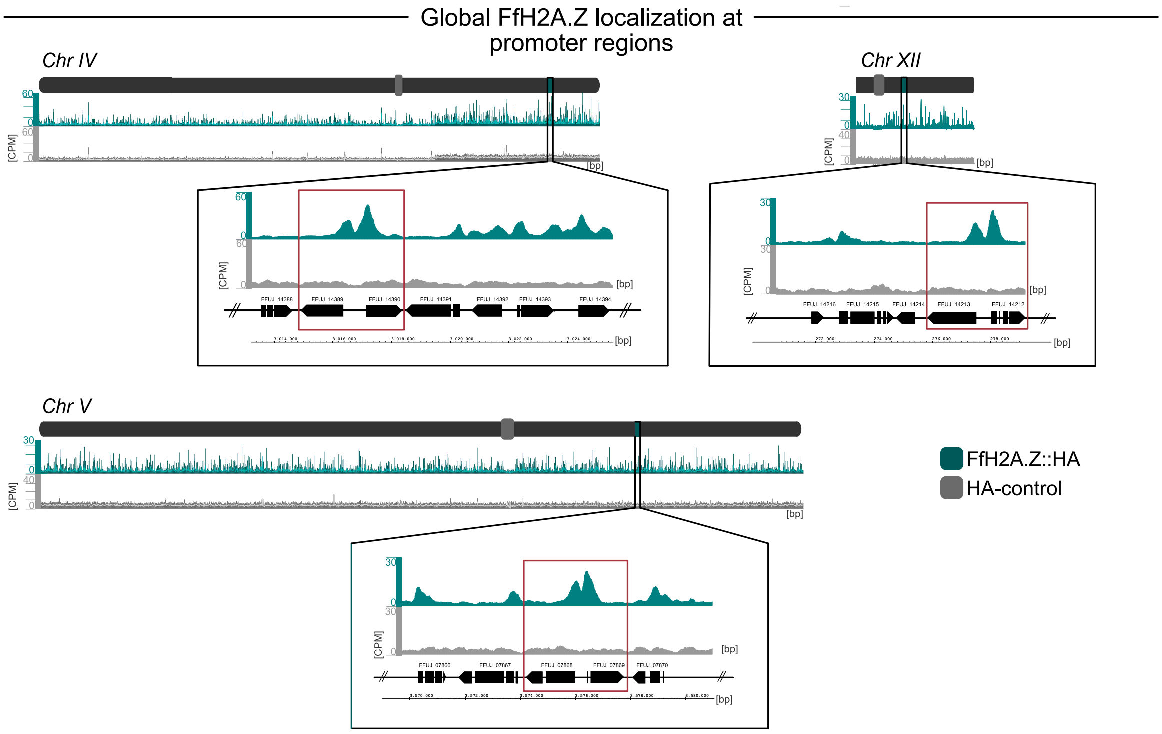

Fig S5 Positioning of the histone variant FfH2A.Z at random chosen loci on chromosome IV, V and XII. Visualization of FfH2A.Z positioning maximal 200 bp upstream and 600 bp downstream surrounding the ATG-sites of random chosen genes. Chromosomes are shown in dark gray, while centromeres are depicted in light gray. Genome-wide distribution of FfH2A.Z present in the *Fusarium fujikuroi* wild type strain is depicted in turquoise and absent from the control (light gray).

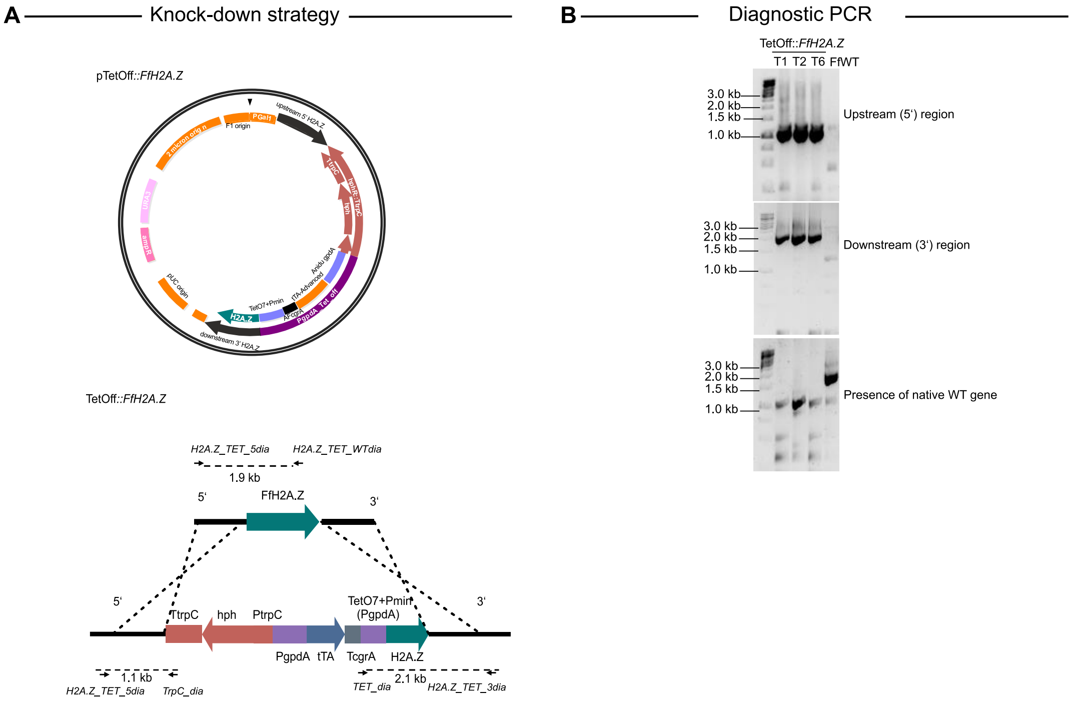

Fig S6 *FfH2A.Z* knock-down strategy and verification. A Plasmid map of the TetOff::*FfH2A.Z* knock-down construct. Before transformation, the plasmid was amplified with the outer primers H2A.Z_TET_5F and H2A.Z_TET_3R. *FfH2A.Z* depletion was approached by the *in locus* exchange of the native promoter against the tetracycline sensitive TetOff promoter system. The verification strategy and primer pairs used for verification of the successful homologous recombination are depicted in the scheme below the plasmid map. **B** Homologous recombination of the construct for three independent knock-down strains TetOff::*FfH2A.Z*_T1, T2 and T6 was determined by diagnostic PCR. Presence of the 5'upstream region was tested using the primer pair H2A.Z_TET_5dia and TtrpC_dia, while integration at the 3'downstream region was tested using the primers H2A.Z_TET_3dia/TET_dia. Absence of the native promoter upstream of *FfH2A.Z* (absence of WT gene) was verified with the primer pair H2A.Z_TET_5dia/H2A.Z_TET_WTdia. As negative (5'/3')/positive (WT gene) control *Fusarium fujikuroi* (FfWT) gDNA was used. As size marker GeneRuler 1 kb Ladder (Thermo Scientific) was used.

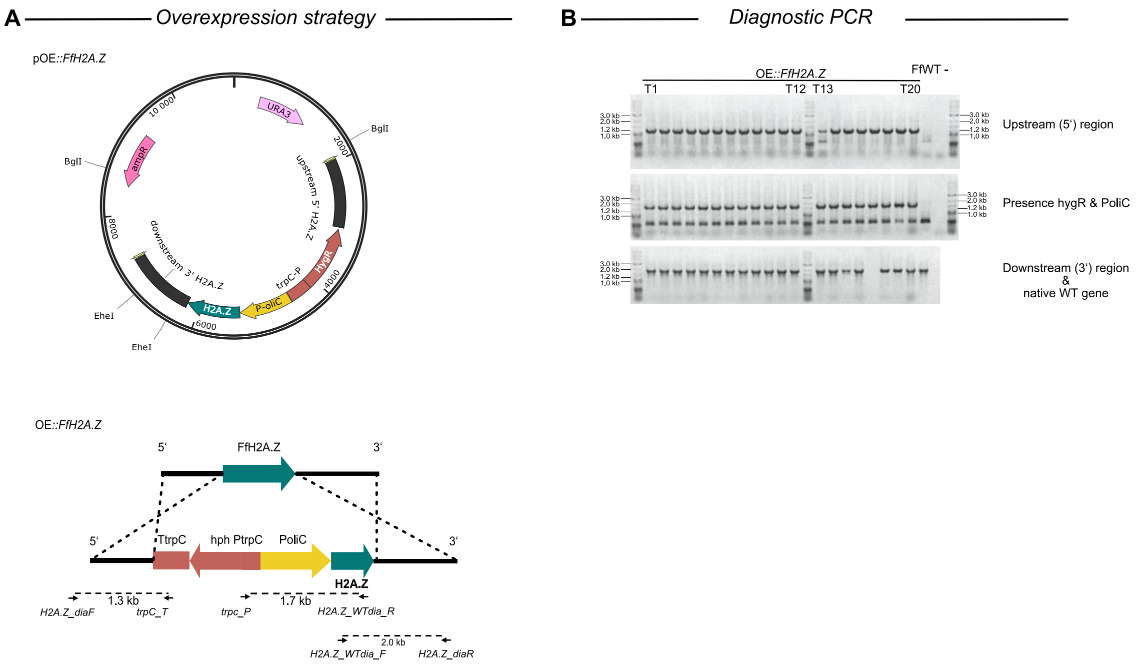

Fig S7 Verification and overexpression strategy of the histone variant *FfH2A.Z*. A Plasmid map of the in locus overexpression (OE::*FfH2A.Z)* construct. Plasmid linearization was performed with the restriction enzymes *Bgl*I and *Ehe*I. The restriction sites are shown in the plasmid map. Constitutive overexpression of *FfH2A.Z* was apporached *via* homologous integration of the strong pOliC promoter from *Aspergillus nidulans* in the native *FfH2A.Z* locus. The verification strategy as well as used primers are visualized in the scheme below the plasmid map. **B** Homologous integration of the constructs was verified *via* diagnostic PCR. The 5′ upstream region and the 3′ downstream region were amplified using the primer pair H2A.ZdiaF/trpC_T and H2A.Z_WTdia_F/H2A.Z_diaR, respectively. Presence of the hygromicin resistence casette and the oliC promoter are tested using the primers trpC_P/H2A.Z_WTdia_R. As negative control *Fusarium fujikuroi* (FfWT) gDNA was used. The 1 kb Plus DNA ladder (NEB) was used as a size marker.

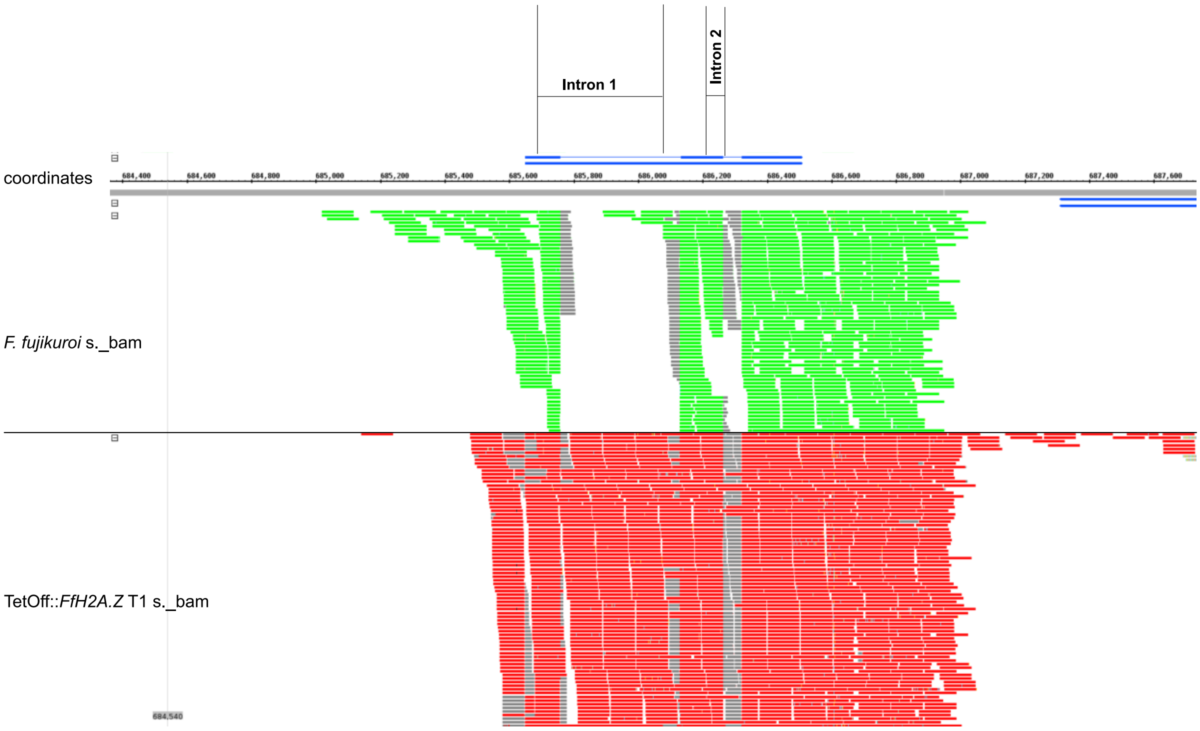

Fig S8 Alternative splicing of *FfH2A*.Z in *Fusarium fujikuroi* (FfWT) and the TetOff::*FfH2A.Z* mutant strain. Bars denote to reads obtained by RNA-seq. The green bars depict the putative two native *FfH2A.Z* splicing isoforms in FfWT, while the red bars show aberrant splicing patterns of the *FfH2A.Z* depletion mutant.

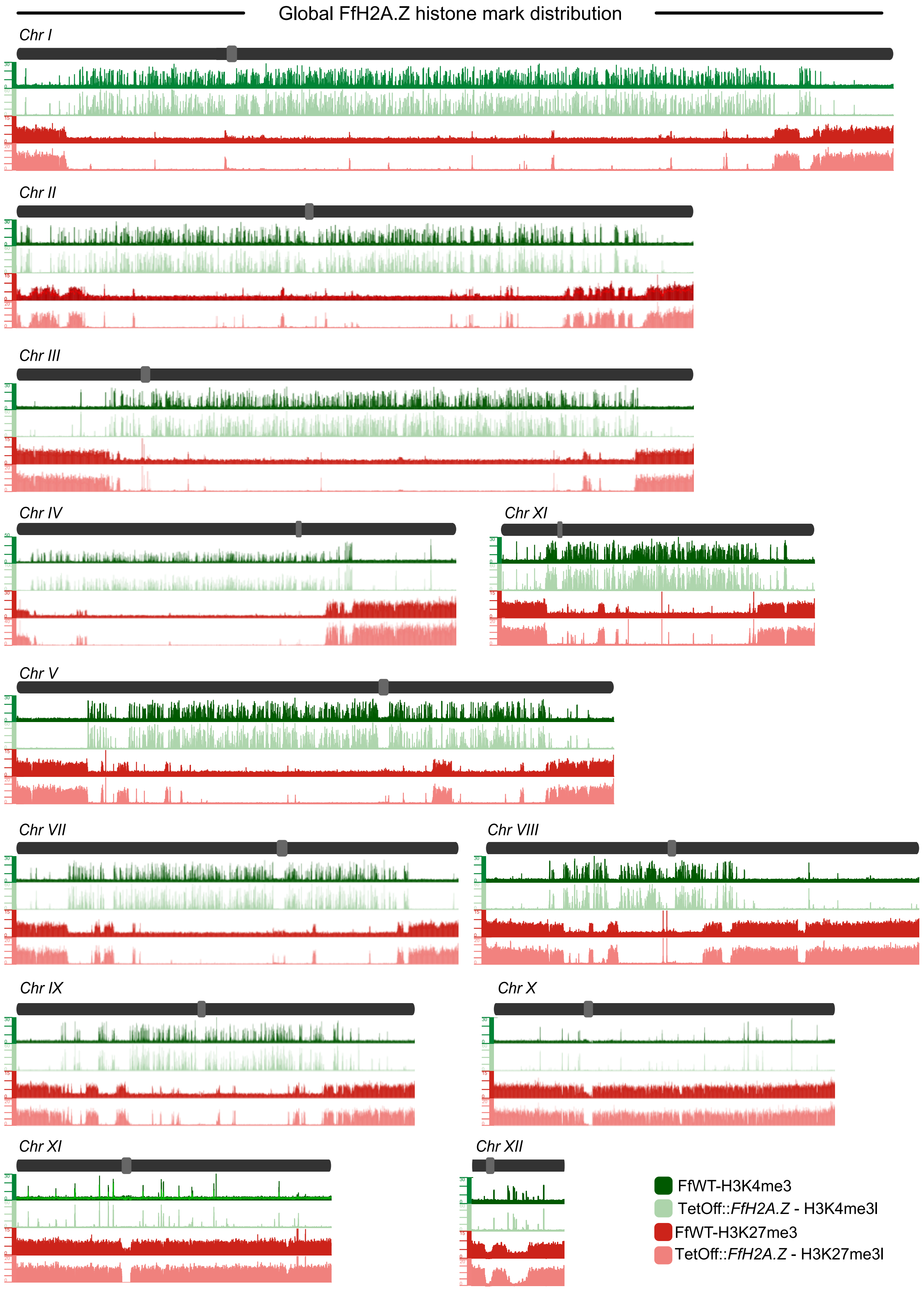

Fig S9 Global distribution of H3K4me3 and H3K27me3 in *Fusarium fujikuroi* (FfWT) and TetOff::*FfH2A.Z.* Chromosomes I-VII are shown in dark gray and centromeres are depicted in light gray. The global distribution of trimethylated H3K4 is shown in green and light-green for FfWT and for the FfH2A.Z depletion mutant, respectively.

The global view on facultative heterochromatin (H3K27me3) in FfWT is depicted in red, while the TetOff::*FfH2A.Z* mutant strain is shown in light red.

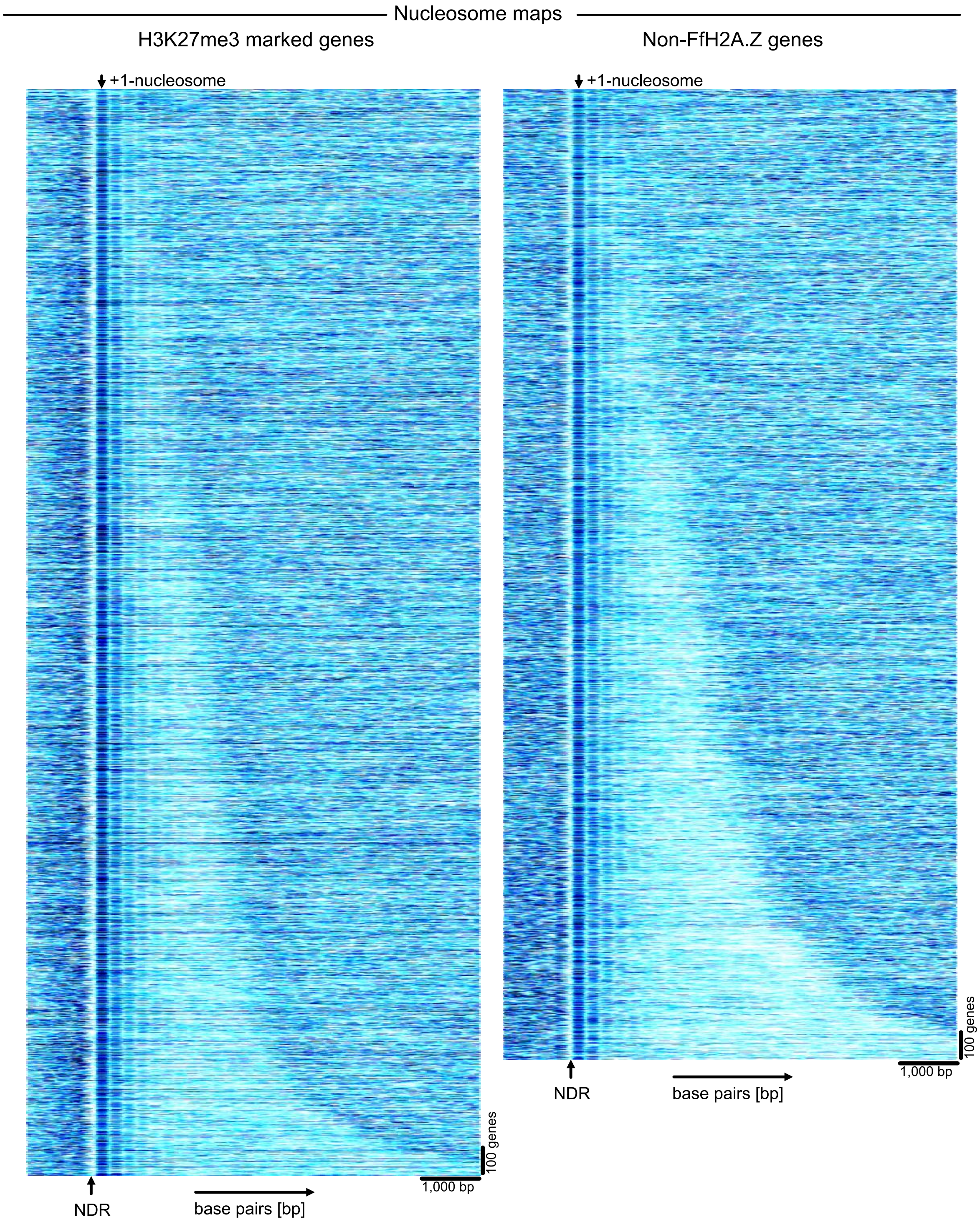

Fig S10 Nucleosome maps of all H3K27me3 genes and non FfH2A.Z genes in *Fusarium fujikuroi* (FfWT). Left panel shows a nucleosome map of all genes marked with H3K27me3, while on the right panel the overall nucleosome distribution of all non-FfH2A.Z genes is depicted. Genes are sorted by increasing gene length top to bottom, arrows top and bottom indicate the position of the +1-nucleosome and the nucleosome depleted regions.

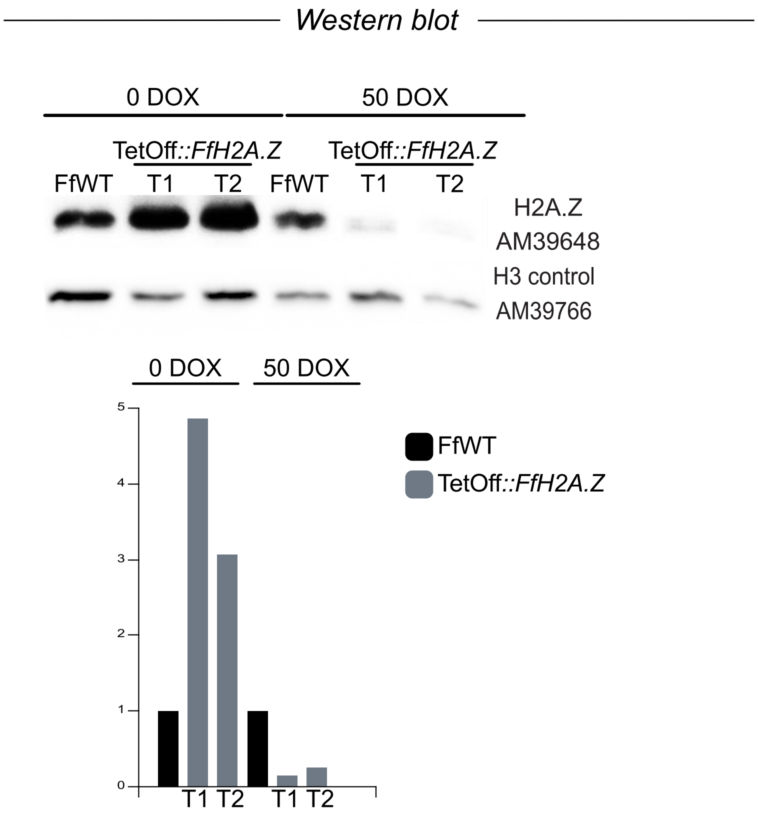

Fig S11 Verification of *FfH2A.Z* silencing in liquid culture after 7 days of incubation. Western blot analysis of *Fusarium fujikuroi* (FfWT) and the FfH2A.Z knock-down (TetOff::*FfH2A.Z*) mutant strains with (50 µg/mL) and without supplementation of the inducing reagent doxycycline (DOX). Proteins were probed with an anti-H2A.Z-specific and an anti H3 C-terminus specific-antibody. For quantification, a densitometric analysis was performed and the FfWT wild-type strain was arbitrarily set to 1.

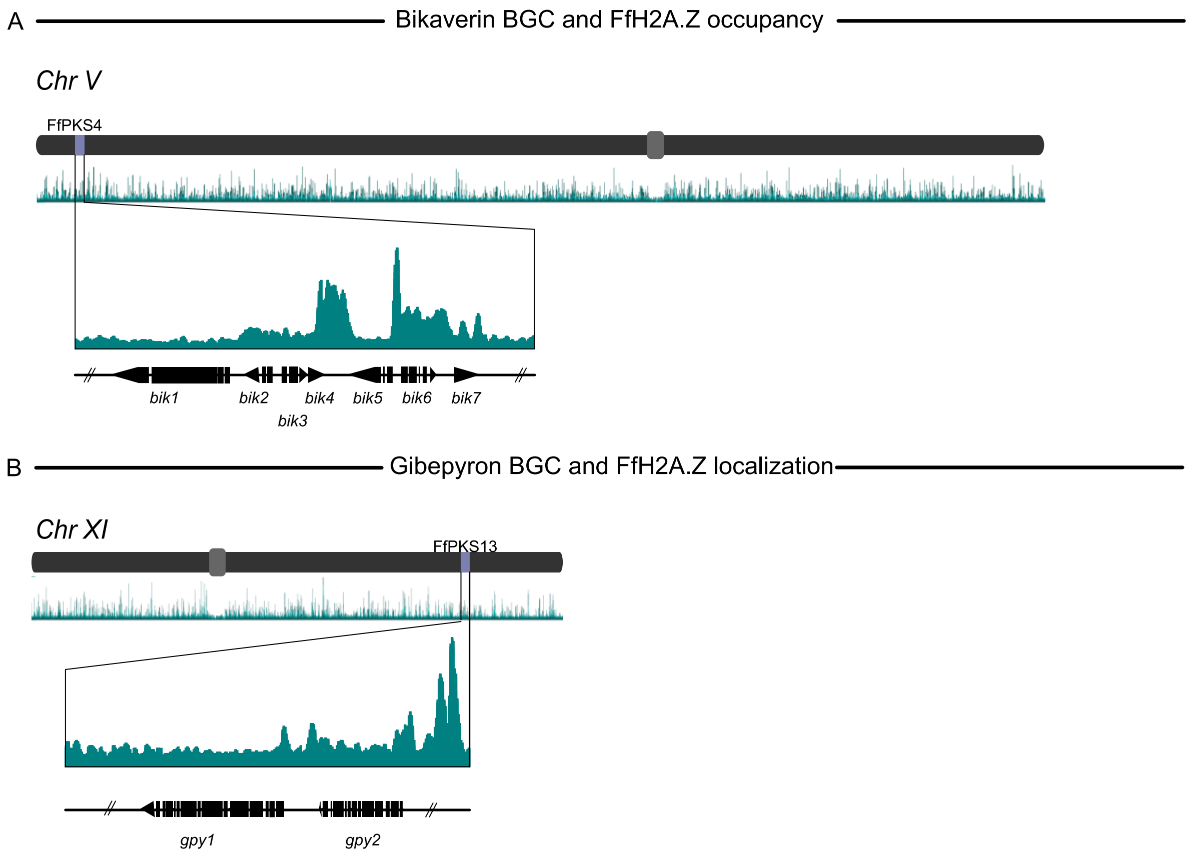

Fig S12 FfH2A.Z occupancy in selected *Fusarium fujikuroi* (FfWT) biosynthetic gene clusters (BGCs). **A** Graphical depiction of the bikaverin (BIK) BGC on chromosome V in FfWT accompanied by the FfH2A.Z distribution. Chromosome V is shown in dark gray and the centromere in light gray. FfH2A.Z distribution is depicted in turquoise. FfPKS4, responsible for the biosynthesis of the pigment BIK is shown in blue. **B** FfH2A.Z distribution within the gibepyrone (GPY) BGC on chromosome XII. The chromosome is shown in dark gray, while the cenrtromere is depicted inlight gray. FfH2A.Z distribution is shown in turquiose. The GPY key-enzyme (FfPKS13) is depicted in blue.

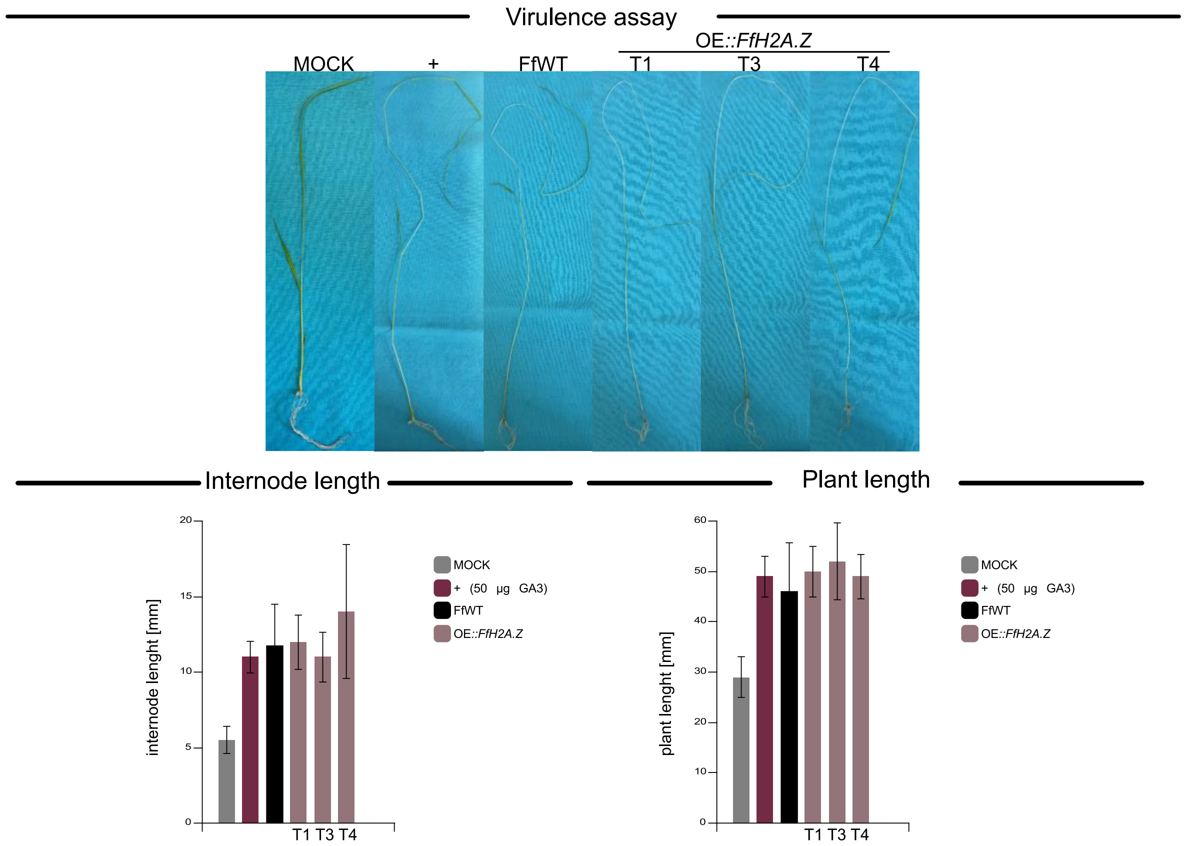

Fig S13 Infection assay on *Oryza sativa* using the FfH2A.Z overexpression (OE::*FfH2A.Z*) strain. The disease progress and typical symptomes were assessed 10 days post inoculation.

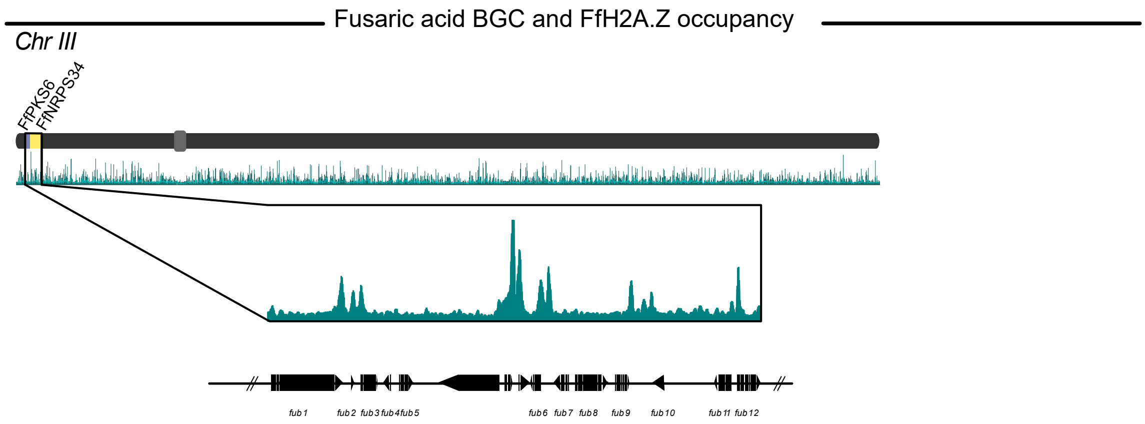

Fig S14 Positioning of the histone variant FfH2A.Z in the fusaric acid (FUB) biosynthetic gene cluster (BGC) in *Fusarium fujikuroi (*FfWT) under non-inducing conditions. The FUB BGC is located on chromosome III, which is is shown in dark grey and the centromere in light grey. FfH2A.Z distribution is depicted in turquoise. FfPKS6 and FfNRPS34, involved in the biosynthesis of FUB are shown in blue and yellow, respectively.

Table S1 Transcriptome of TetOff::*FfH2A.Z* and OE::*FfH2A.Z* in comparison to the *F. fujikuroi* wild-type strain (FfWT).

Table S2 Gene Ontology (GO) enrichment analysis of down-regulated genes enriched with FfH2A.Z of biological processes.

Table S3 Gene Ontology (GO) enrichment analysis of up-regulated genes enriched with FfH2A.Z of biological processes.

Table S4 Table of conserved gene analysis including FfH2A.Z marked genes and genes decorated with the histone marks H3K27me3 and/or H3K4me3.

Table S5 Overview of primers used in this work. Introduced overhangs required for cloning are written in uppercase letters.

Gene ID	Primer ID	Primer sequence
**Primers for plasmid construction and fragment amplification**		
**FFUJ_13657** **TetOff::*FfH2A.Z***	H2A.Z_TET_5F	GTAATACGACTCACTATAGGGAATATTcttggccattggcaagtgtgg
	H2A.Z_TET_5R	GAATGCACAGGTACACTTGTTTAGAGGtgttgatatgatcgcgtaa
	H2A.Z_TET_3F	TTGAGCAGACATCACCGTTTAAACACCatgcctggtggaaagggaaag
	H2A.Z_TET_3R	GACATAACTAATTACATGATGCGGCCCccaagctgtggtcgtgcgg
	hph_PgpdA_F	AATGCTCCTTCAATATCATCTTCTGTCcggagaatatggagcttcatc
	TET_off_R	ggtgtttaaacggtgatgtc
	hph_F	gacagaagatgatattgaaggagc
	TtrpC_R	cctctaaacaagtgtacctg
**FFUJ_13657** **OE::*FfH2A.Z***	OE-H2A.Z_5F	ACGCCAGGGTTTTCCCAGTCACGACGagggaagcgacgaggaacaatggc
	OE-H2A.Z_5R	AACCAGTTAACGTTAACCCCGGGcggtagccgatcggtcgaaatggc
	OE-H2A.Z_GenF	CCATCACATCACAATCGATCCAACCATGCCTGGTGGAAAGGGAAAGTC
	H2A.Z_YRC_3R	ATAACAATTTCACACAGGAAACAGCtggtgcgcatgcttgagaggaagc
**FFUJ_13657** ***FfH2A.Z*::HA**	Primer-3R	gctgtttcctgtgtgaaattgttatccgc
	HiFi-AmpR-p5-R	tgactgggaaaaccctggcgtCAGGTGGCACTTTTCGGGGAAATG
	HiFi-H2AZ-HU-5F	ACGCCAGGGTTTTCCCAGTCACGACGagggaagcgacgaggaacaatg
	HiFi_C-HA-H2A.Z_R	TCAGGAACATCGTATGGGTAgccttcaagtgccttggccttc
	3xHA	tacccatacgatgttcctgactatgcgggctatccctatgacgtcccggactatgcaggatcctatccatatgacgttccagattacgct
	C-HA-Tgluc_F	CATATGACGTTCCAGATTACGCTtaacgtatgtagataagatgtatgattaggggttg
	HPH-F	gtcggagacagaagatgatattgaaggagc

	HiFi-H2AZ-HD-pTrpc	TTCAATATCATCTTCTGTCTCCGACgcggtaaaccggtatatgggatgg
	HiFi-H2AZ-HD-3R	ATAACAATTTCACACAGGAAACAGCtggtgcgcatgcttgagagg
**FFUJ_13657** **HA::*FfH2A.Z***	Primer-3R	gctgtttcctgtgtgaaattgttatccgc
	HiFi-AmpR-p5-R	tgactgggaaaaccctggcgtCAGGTGGCACTTTTCGGGGAAATG
	HiFi-H2AZ-HU-5F	ACGCCAGGGTTTTCCCAGTCACGACGagggaagcgacgaggaacaatg
	HiFi-H2A.Z-BH_R	AGGAACATCGTATGGGTACATtgttgatatgatcgcgtaaaaaaaatcg
	3xHA	tacccatacgatgttcctgactatgcgggctatccctatgacgtcccggactatgcaggatcctatccatatgacgttccagattacgct
	HiFi-H2A.Z-HHA_F	GACGTTCCAGATTACGCTatgcctggtggaaagggaaagtcttctggc
	H2A.Z-BcTgluc_R	TCATACATCTTATCTACATACGttagccttcaagtgccttggcc
	Tgluc-F2	cgtatgtagataagatgtatg
	HPH-F	gtcggagacagaagatgatattgaaggagc
	HiFi-H2AZ-HD-pTrpc	TTCAATATCATCTTCTGTCTCCGACgcggtaaaccggtatatgggatgg
	HiFi-H2AZ-HD-3R	ATAACAATTTCACACAGGAAACAGCtggtgcgcatgcttgagagg
**Primers used for diagnostic PCR**		
**FFUJ_13657**	H2A.Z_diaF	aagcagcagagtcactctgc
	H2A.Z_diaR	ccttcttcttctgctcgacc
	H2A.Z_diaTagF	tgtacctctgtgccatccaatcc
	H2A.Z_diaTagR	catgtgcttattgaaagcgcagg
	H2A.Z_WTdia_F	aagcagcagagtcactctgc
	H2A.Z_WTdia_R	ccttcttcttctgctcgacc
	H2A.Z_TET_5F	GTAATACGACTCACTATAGGGAATATTcttggccattggcaagtgtgg

**FFUJ_13657**	H2A.Z_TET_5R	GAATGCACAGGTACACTTGTTTAGAGGtgttgatatgatcgcgtaa
	H2A.Z_TET_WTdia	ctgcttcttggtgccctcagc
/	trpC_T	ggaatagagtagatgccgaccgg
/	trpc_P	cctccactagctccagccaagccc
/	trpc_P2	gtgatccgcctggacgactaaacc
/	Bcgluc_seqR	gggtccatgctaatacttatgtac
/	Tgluc_hiF	catacgtacatctgatttgacaacc
/	TtrpC_dia	cagaatgcacaggtacacttg
/	TET_dia	ccatccttcccatcccttattcc
**Primers used for sequencing**		
/	pRS426_seqF	gccattcaggctgcgcaactg
/	hph-hiF	gtctggaccgatggctgtgtagaag
/	TtrpC-hph_R	cccggggttaacgttaactggttcc
/	ptrpC-hph_F	gatattgaaggagcattttttggg
/	pCSN44-trpC-P2	gtgatccgcctggacgactaaacc
/	pOliC-F2	gaaactcagtctccttgg
/	pRS426_seq_R	gttgtgtggaattgtgagcgg
**Primers for semi-quantitative and RT-qPCR**		
*cDNA verification**		
**FFUJ_02611**	cDNA_check_Actin_F	gtatgtgcaaggccggtttcg
	cDNA_check_Actin_R	gagaccagggtacatggtgg
*Housekeeping genes**		

**FFUJ_02611**	Actin_F	ccaccatgtaccctggtctctcc
	Actin_R	aatggaaccaccgatccagacgg
**FFUJ_07385**	ß-TUB_F	gaggcagtacgatggcatgcg
	ß-TUB_R	ggtaatctgcgtcttcagcagcttcg
**FFUJ_13490**	GPD_F	gcctctgagggtgacctcaagg
	GPD_R	cgttgtcgtaccaggagaccagc
**FFUJ_13657**	RT-qPCR_H2A.Z_F	ctcgagttggcaggtaacgctgc
	RT-qPCR_H2A.Z_R	atgtgtggcaggacaccaccg

REFERENCES

1. Blum M, Chang H-Y, Chuguransky S, Grego T, Kandasaamy S, Mitchell A, et al. The InterPro protein families and domains database: 20 years on. Nucleic Acids Research. 2021;49(D1):D344-D54.
